# Single-nucleus RNA-seq and ATAC-seq analyses provide molecular insights into cadmium-stress response in alfalfa roots

**DOI:** 10.1093/hr/uhag117

**Published:** 2026-04-06

**Authors:** Yuqi Zhang, Hao Liu, Ming Xu, Mengjia Xie, Shuhan Deng, Xinyue Ma, Li Zhao, Fei He, Mingna Li, Ruicai Long, Xue Wang, Junmei Kang, Qingchuan Yang, Lin Chen

**Affiliations:** Institute of Animal Science, Chinese Academy of Agricultural Sciences, Beijing 100193, China; Institute of Animal Science, Chinese Academy of Agricultural Sciences, Beijing 100193, China; Institute of Animal Science, Chinese Academy of Agricultural Sciences, Beijing 100193, China; Glbizzia Biosciences Co., Ltd., Beijing 102609, China; Glbizzia Biosciences Co., Ltd., Beijing 102609, China; Institute of Animal Science, Chinese Academy of Agricultural Sciences, Beijing 100193, China; Institute of Animal Science, Chinese Academy of Agricultural Sciences, Beijing 100193, China; Institute of Animal Science, Chinese Academy of Agricultural Sciences, Beijing 100193, China; Institute of Animal Science, Chinese Academy of Agricultural Sciences, Beijing 100193, China; Institute of Animal Science, Chinese Academy of Agricultural Sciences, Beijing 100193, China; Institute of Animal Science, Chinese Academy of Agricultural Sciences, Beijing 100193, China; Institute of Animal Science, Chinese Academy of Agricultural Sciences, Beijing 100193, China; Institute of Animal Science, Chinese Academy of Agricultural Sciences, Beijing 100193, China; Institute of Animal Science, Chinese Academy of Agricultural Sciences, Beijing 100193, China

## Abstract

Soil cadmium (Cd) pollution threatens global food security. Elucidating plant cellular responses to Cd stress is critical for the breeding of low-Cd-accumulating crops. Here, we investigated Cd-responsive regulatory mechanisms in alfalfa at single-cell resolution and identified key Cd-associated genes. Eight major root cell types were annotated, with significant remodeling under Cd stress. Under Cd stress, root endodermal and phloem cells adopt distinct adaptive strategies: endodermal cells shift toward a Cd sequestration and detoxification state, whereas phloem cells exhibit a response gradient ranging from basic defense to systemic regulation. Integrated multiomics analyses revealed cell-type-specific genome-wide changes in chromatin accessibility, which positively correlated with gene expression—particularly in promoter regions. Key genes including *MsGSH1*, *MsMT2A*, *MsHMP47*, and *MsABCC3* were shown to increase Cd tolerance in yeast. Coexpression network analysis revealed 10 cell-type-specific modules, with the calmodulin-like gene *MsCML* acting as a highly interconnected hub gene, whose overexpression significantly improved Cd tolerance. These findings provide valuable genetic resources and a theoretical basis for the precise breeding of low-Cd-accumulating forage, with implications for understanding Cd-responsive epigenetic and transcriptional regulation in plants.

## Introduction

As industrialization and urbanization advance, heavy metal pollution in soil is becoming an increasingly severe global issue, particularly because of activities from the mining sector. Cadmium (Cd) stands out among the heavy metals because of its high toxicity to human health [1]. This nonessential element is highly mobile and readily accumulates in plants via root absorption, disrupting critical metabolic pathways such as photosynthesis and respiration [2]. Moreover, Cd can bioaccumulate in the food chain and thereby in the human body, posing serious health risks [3]. Therefore, elucidating the molecular mechanisms underlying plant responses to Cd stress is essential for the development of new Cd-tolerant crop varieties [4].

Recent research has revealed a diverse array of genes that govern plant responses to Cd stress, which orchestrate defense mechanisms such as uptake, transport, sequestration, detoxification, and signal transduction. Specifically, genes such as *IRT1* (iron-regulated transporter 1) [5], *ZIP2* [6], *ZIP3* [7], and *ZIP7* [8] (zinc-regulated transporter-like proteins) are integral to the initial uptake and subsequent transport of Cd within plants. Similarly, natural-resistance-associated macrophage proteins (NRAMPs) such as *OsNRAMP1* [9] and *OsNRAMP5* [10] in rice and *AtNRAMP6* [11] in Arabidopsis facilitate Cd uptake and redistribution.

In terms of sequestration and detoxification, *HMA2* [12] and *HMA3* (heavy metal ATPases) [13] function as transporters that sequester Cd into vacuoles, thereby reducing its cytotoxicity. The *ABCG36* [14] and *AtPDR8* [15] (pleiotropic drug resistance) genes, both of which belong to the ABC transporter family, act as Cd efflux pumps, actively exporting Cd out of the cell. The *AtABCC1*, *AtABCC2* [16], and *AtABCC3* [17] genes further contribute to detoxification by transporting Cd-glutathione (GSH) complexes into vacuoles. Phytochelatin synthesis, mediated by the phytochelatin synthase (PCS) gene, represents a pivotal detoxification pathway [18]. Phytochelatins chelate Cd ions, forming complexes that are subsequently sequestered into vacuoles. The *GSH1* and *GSH2* genes are essential for the biosynthesis of GSH [19], a key player in Cd detoxification [20]. The glutathione reductase (GR) gene maintains the redox balance of GSH [21], ensuring its availability for Cd detoxification. Signaling pathways activated under Cd stress involve transcription factors (TFs) such as *WRKY45* [22], which modulate the expression of stress-responsive genes. In maize, *ZmMPK3*-1 and *ZmMPK6*-1 [23] (mitogen-activated protein kinases) are activated by reactive oxygen species (ROS) generated in response to Cd exposure, initiating downstream signaling cascades. The *TaEXPB23* [24] gene in wheat encodes an expansion protein that enhances Cd tolerance by facilitating cell wall modifications. Additional genes such as *OsHMA2* [25], *OsLCT1* [26] (low-Cd accumulation 1), *CAL1* [27] (Cd low accumulator 1), and *OsCCX2* [28] (calcium and Cd exchanger 2) have been identified as key contributors to Cd tolerance in rice. These genes collectively underscore the complex genetic architecture underlying Cd tolerance in plants, providing valuable insights for the development of Cd-tolerant crop varieties through molecular breeding and genetic engineering strategies.

Single-cell or single-nucleus RNA sequencing (scRNA-seq or snRNA-seq) has improved our understanding of the regulatory mechanisms underlying plant growth, development, and environmental adaptation. Numerous scRNA-seq atlases have been developed for various tissues in different plants, such as those related to root development in Arabidopsis [29], rice [30], and maize [31]; leaf development in peanut [32]; and stem development in poplar [33, 34]. In studies on plant responses to Cd stress, scRNA-seq has been used to explore dynamic gene expression in Arabidopsis under Cd stress [35], with a focus on the mechanisms of action of xylem-specific genes such as *AHP1*, *DOF2.4*, *NHL1*, *CDF4,* and *ATARFB1A* [36]. In sedum (stonecrop), scRNA-seq revealed cell-type-specific transcriptional regulation and confirmed the existence of a unique gene regulatory network that responds to Cd stress [37]. Recent studies have expanded this approach to include single-cell chromatin accessibility analysis (snATAC-seq). Compared with scRNA-seq, which primarily captures mature mRNA, snRNA-seq effectively circumvents challenges such as difficult digestion of plant cell walls, cellular stress induction, and interference from secondary metabolites. Therefore, this study employs snRNA-seq to more accurately and comprehensively elucidate the underlying biological mechanisms and genotype–phenotype associations. [38]. Compared with the substantial research in animals and humans using combined snRNA-seq and snATAC-seq analyses, fewer studies in plants have applied these approaches, and the existing work has been focused mainly on growth and development. In terms of plant development, combined snRNA-seq and snATAC-seq analyses have revealed cell-type-specific regulatory networks involved in the development of various tissues in maize [39], soybean [40], rice [41], wheat [42], and peanut [43]. Compared with studies on plant growth and development, fewer studies have focused on plant responses to abiotic stresses. The mechanisms underlying Arabidopsis root responses to osmotic stress have been elucidated using snRNA-seq and snATAC-seq technologies [44]. An analysis of the differences in osmotic stress responses among various cell types in the root tip revealed that root hairs are more sensitive to osmotic stress. In addition, snRNA-seq and snATAC-seq technologies have been used to elucidate the mechanisms underlying Chinese cabbage root responses to salt stress [45] and cotton anther responses to high-temperature stress [46]. However, no reports have yet been published on the use of these technologies to study plant responses to heavy metals.

As one of the most important forage crops globally, alfalfa (*Medicago sativa* L.) is renowned as the ‘King of Forages’ because of its high nutritional value [47]. Cd stress negatively affects alfalfa yield and quality [48]. Cloning genes from alfalfa is generally challenging because of its allotetraploid nature and self-incompatibility [49], which further complicates efforts to identify genes related to Cd stress. To date, only a limited number of Cd-stress-responsive genes have been successfully cloned from alfalfa. For instance, the *MsYSL6* gene encodes a yellow stripe-like transporter protein [50], and its overexpression confers Cd tolerance in transgenic tobacco. The *MsHIPP12* gene encodes a heavy-metal-associated protein [51], the expression of which is induced by various abiotic stresses. The overexpression of *MsHIPP12* significantly increases Cd tolerance in transgenic *Arabidopsis thaliana* [51]. In our previous research, we conducted a combined analysis of DNA methylation, RNA methylation, and transcriptome sequencing of alfalfa roots under Cd stress [52]. We discovered that Cd stress reduced DNA methylation levels while increasing RNA methylation levels. In yeast, the overexpression of two genes—*MsNARMP5*, which is modified by DNA methylation, and *MsPCR2*, which is modified by RNA methylation—significantly improved Cd tolerance. However, traditional bulk RNA sequencing provides only an average value of gene expression abundance across all cells within a tissue and fails to reveal the differences in the transcriptional regulation of key genes in different cell types under Cd stress. Thus, this technique masks crucial cell-specific transcriptional regulatory information. Moreover, the relationship between chromatin accessibility across the entire genome and gene transcriptional regulation in different cell types under Cd stress in alfalfa remains unclear.

In this study, we integrated snRNA-seq and snATAC-seq to elucidate the changes in gene expression abundance in different cell types of alfalfa roots under Cd stress and their relationships with chromatin accessibility and to identify key genes related to the cadmium-stress response. Our findings provide valuable insights into the molecular regulatory network of plant root responses to Cd stress and offer important genetic resources for the future development of cadmium-tolerant alfalfa varieties.

## Results

### Cd stress had a substantial negative effect on the growth and development of alfalfa seedlings

**Figure 1:**
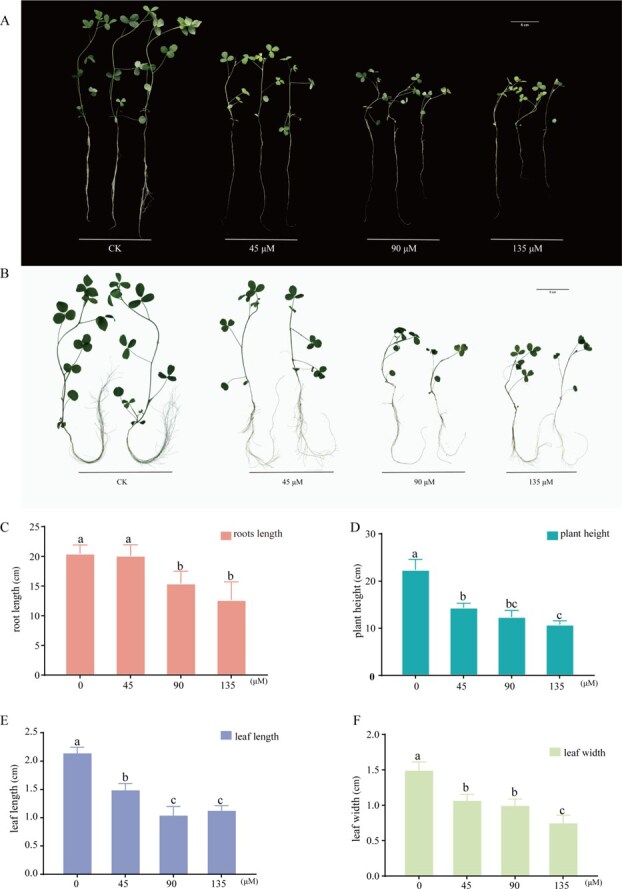
Phenotypic analysis of alfalfa under Cd stress. (A) Representative photographs of alfalfa plants exposed to varying concentrations of CdCl₂, showing clear dose-dependent growth inhibition. Bar = 1 cm. (B) Corresponding high-resolution scans of root systems illustrating Cd-induced alterations in root architecture. Bar = 1 cm. (C–F) Quantitative assessment of key growth parameters: (C) root length, (D) plant height, (E) leaf length, and (F) leaf width. Data are presented as the mean ± SE (*n* ≥ 3 biological replicates). Different lowercase letters denote statistically significant differences among treatments (one-way ANOVA followed by the least significant difference (LSD) test, *P* < 0.05).

Different concentrations of Cd chloride (CdCl_2_) significantly inhibited the growth of alfalfa seedlings in a concentration-dependent manner. Three-week-old seedlings were treated with various concentrations (0, 45, 90, and 135 μM) of CdCl_2_ solution for 5 days, after which their growth parameters were statistically evaluated. As shown in Fig. 1A and B, Cd stress markedly suppressed overall seedling development. Key growth metrics, including root length, leaf length, leaf width, and plant height, tended to decrease with increasing Cd concentration (Fig. 1C–F). Specifically, under the 90 μM Cd treatment, compared with those under the control treatment, the root length decreased by 30%, the leaf width decreased by 28%, and the plant height declined by 35% compared to the control (all comparisons *P <* 0.05). These findings indicate that Cd toxicity directly interferes with normal seedling growth. Furthermore, both leaf elongation and expansion were significantly inhibited, impairing the development of aboveground photosynthetic organs. Hyperspectral imaging data further confirmed that even low concentrations of Cd significantly affected leaf photosynthetic function (Fig. S1, Table S13). In summary, Cd stress disrupts multiple physiological processes, ultimately leading to overall impairment of biomass accumulation and morphological development in alfalfa seedlings.

### Cd stress affects multiple metabolic processes in alfalfa roots

**Figure 2:**
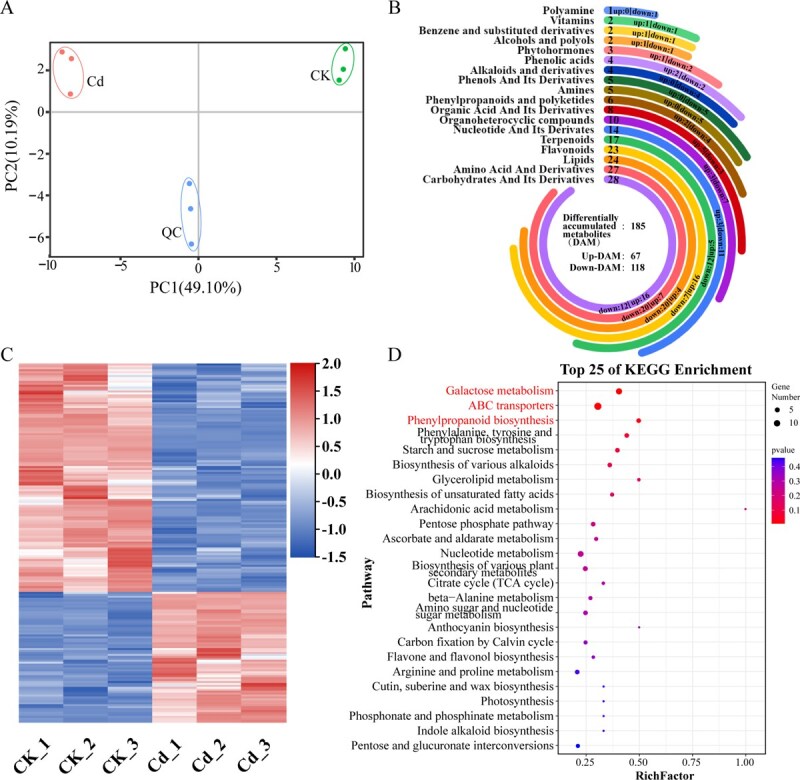
Metabolomic profiling reveals comprehensive reprogramming in alfalfa roots under Cd stress. (A) PCA score plot demonstrating clear separation between control, Cd-treated, and blank samples, indicating distinct metabolic states. (B) Bar chart summarizing the number and categories of metabolites whose abundance significantly differed DAMs in response to Cd stress. (C) Hierarchical clustering heatmaps of DAMs. (D) KEGG pathway enrichment analysis of the DAMs, highlighting the most significantly impacted metabolic pathways.

To thoroughly investigate the impact of Cd stress on the metabolic processes of alfalfa roots, we conducted metabolomics analysis on root tissues under Cd stress (90 μM for 5 days) and under normal conditions. The results revealed a total of 918 metabolites in the roots of alfalfa (Fig. S2). Principal component analysis (PCA) indicated that Cd stress significantly affected metabolic processes in alfalfa roots (Fig. 2A). Under Cd stress, a total of 185 differentially abundant metabolites (DAMs) were identified, including 67 upregulated and 118 downregulated metabolites (Table S2). These DAMs were classified into 18 different categories, among which 28 were derived from carbohydrates, 27 were derived from amino acids, 24 were derived from lipids, and 23 were derived from flavonoids (Fig. 2B and C). These four categories of DAMs constituted more than 55% of the total DAMs. Among the upregulated DAMs, carbohydrates and their derivatives and flavonoids were the most abundant, each with 16 compounds, accounting for nearly half of all upregulated DAMs (Fig. S3A). In the classification of downregulated DAMs, five categories had more than 10 metabolites, with amino acids and their derivatives and lipids being the most numerous, each with 20 compounds, followed by carbohydrates and their derivatives and terpenoids, each with 12 compounds, and nucleotides and their derivatives with 11 compounds (Fig. S3B). These results demonstrated that Cd stress significantly affected metabolic processes in alfalfa roots.

Further Kyoto Encyclopedia of Genes and Genomes (KEGG) enrichment analysis of the DAMs revealed a total of 60 pathways, covering four major categories: metabolism (56 pathways), genetic information processing (two pathways), environmental information processing (one pathway), and cellular processes (one pathway). Among all the enriched pathways, three pathways were significantly enriched: ‘galactose metabolism’, ‘ABC transporters’, and ‘phenylpropanoid biosynthesis’ (Fig. 2D). Analysis of the upregulated DAMs revealed enrichment in 37 pathways, among which four pathways were significantly enriched: ‘galactose metabolism’, ‘starch and sucrose metabolism’, ‘fatty acid metabolism’, and ‘ABC transporters’ (Fig. S3C). In the functional analysis, all the downregulated DAMs were enriched in 44 pathways, with only the ‘nucleotide metabolism’ pathway being significantly enriched (Fig. S3D). A comprehensive analysis of the functional results of upregulated and downregulated DAMs revealed that pathways related to carbohydrate compounds were significantly enriched, especially for upregulated metabolites, indicating that the synthesis and metabolism of carbohydrate compounds play important roles in the response of alfalfa to Cd stress.

### Construction of a single-cell atlas and identification of cell types in alfalfa roots under normal and Cd-stress conditions

**Figure 3:**
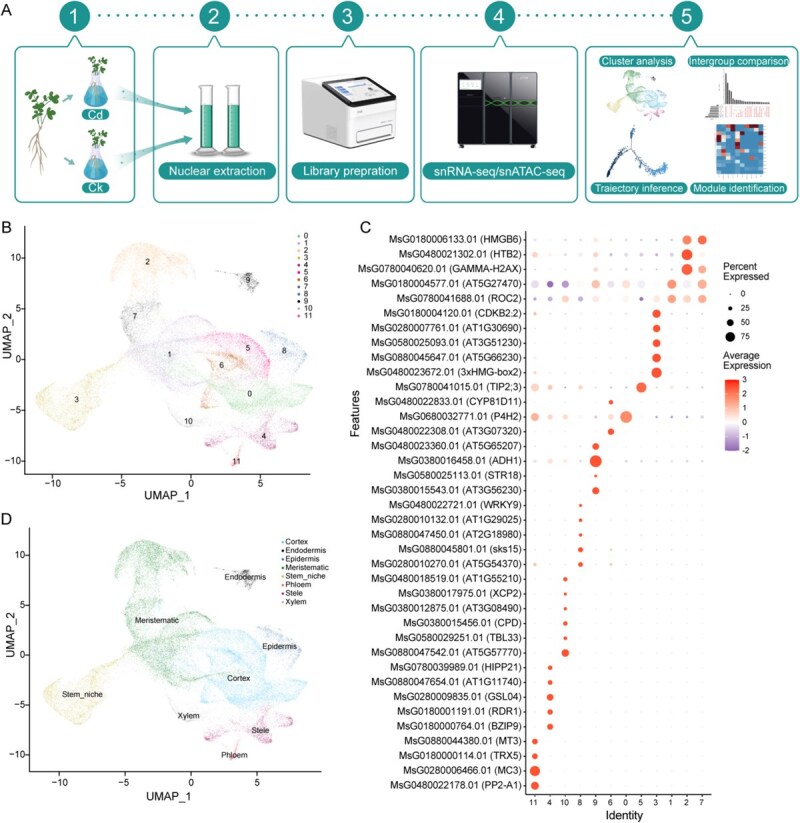
Construction of a single-nucleus multiomics atlas for identifying alfalfa root cell types. (A) Schematic workflow outlining the experimental procedure from sample preparation and nuclear isolation to snRNA-seq/snATAC-seq library construction and integrated data analysis. (B) UMAP projection of 11 distinct clusters identified from the snRNA-seq data. (C) Dot plot displaying the expression of 38 well-established cell-type-specific marker genes used for annotating the clusters. Dot size represents the percentage of cells expressing the gene. (D) UMAP visualization of the eight annotated cell types based on marker gene expression.

To construct a high-quality single-cell atlas of alfalfa, we conducted both snRNA-seq and snATAC-seq on nuclei isolated from roots of 1-cm-long root tips under 90 μM CdCl_2_ for 5 days (Cd stress) and normal conditions (CK), respectively. Three biological replicates were performed for each sample (Fig. 3A). After the aberrant cells were filtered, a total of 44 894 high-quality nuclei were obtained from the control and Cd-stress samples. The median number of unique molecular identifiers (UMIs) per cell was 1311 in the control samples and 949 in the Cd-stress samples. The median number of genes detected per cell was 822 in the control samples and 698 in the Cd-stress samples. In addition, a total of 65 992 nuclei were retained for snATAC-seq, yielding an average of 1337 fragments per nucleus. High concordance between biological replicates was found for our single-cell transcriptome and ATAC data (Fig. S4, Fig. S16).

To understand the cell types in alfalfa roots, the snRNA-seq data were subjected to PCA for dimensionality reduction and subsequent graph-based clustering. A total of 12 unique cell clusters were identified in both the control and Cd-stress samples (Fig. 3B). The clusters were consistently present across conditions, allowing for integrated analysis. Following cluster identification, cell type annotation was performed using a set of canonical marker genes. To this end, we leveraged orthologous relationships and protein domain conservation to map known markers from *A. thaliana* and *Medicago truncatula* to the alfalfa genome (Table S1) [2–4, 37]. This approach led to the annotation of eight distinct cell types (Fig. 3C).

Specifically, cortical cells were characterized by *P4H2* (Cluster 0), *TIP2;3* (Cluster 5), and *CYP81D11* and *AT3G07320* (Cluster 6). Endodermal cells were identified by *STR18*, *ADH1*, *AT3G56230*, and *AT5G65207* (Cluster 9). Epidermal cells were defined as *SKS15*, *WRKY9*, *AT5G54370*, *AT2G18980*, and *AT1G29025* (Cluster 8). Meristematic cells were marked by *ROC2* and *AT5G27470* (Cluster 1), *GAMMA-H2AX* and *HTB2* (Cluster 2), and *HMGB6* (Cluster 7). Stem-niche cells were annotated on the basis of genes involved in the cell cycle and stem cell maintenance, including *CDKB2;2* and *HMGB6* (Cluster 3). Phloem cells were distinguished by *PP2-A1*, *MC3*, *TRX5*, and *MT3* (Cluster 11). Stele cells were characterized by *BZIP9*, *RDR1*, *GSL04*, *HIPP21*, and *AT1G11740* expression (Cluster 4). Xylem cells were identified using *TBL33*, *CPD*, *XCP2*, *AT1G55210*, *AT5G57770*, and *AT3G08490* (Cluster 10) (Fig. S5). The orthologous genes used as markers to annotate the remaining cell types are detailed in Table S1.

In summary, eight cell types were identified on the basis of the expression patterns of the marker genes (Fig. 3D). They included cortex cells (14 609 cells; Clusters 0, 5, and 6), endodermis cells (1143 cells; Cluster 9), epidermis cells (1937 cells; Cluster 8), meristematic cells (16 979 cells; Clusters 1, 2, and 7), stem-niche cells (5399 cells; Cluster 3), phloem cells (602 cells; Cluster 11), stele cells (3214 cells; Cluster 4), and xylem cells (1011 cells; Cluster 10).

### Transcriptional response of alfalfa roots to Cd stress at the single-cell level

**Figure 4:**
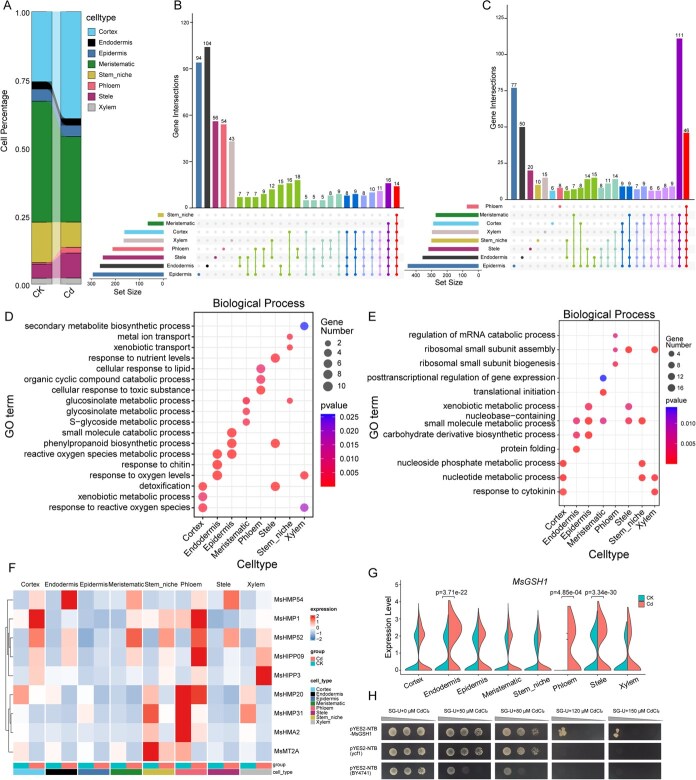
Cell-type-specific transcriptional dynamics in response to cadmium stress. (A) Stacked bar plot showing the proportional change in cell-type abundance between CK and Cd stress. (B and C) UpSet plots illustrating the number of upregulated (B) and downregulated (C) genes that are specific to or shared among different cell types. (D and E) GO enrichment analysis of upregulated (D) and downregulated (E) genes within each cell type cluster. (F) Heatmap depicting the relative expression levels of key genes involved in heavy metal ion transport across all annotated cell types. (G) Violin plots comparing the expression distribution of *MsGSH1* between the control and Cd-treated groups for each cell type. (H) Functional validation assay demonstrating that heterologous expression of *MsGSH1* enhances Cd tolerance in yeast.

Under both control (CK) and Cd stress conditions, eight distinct cell types were identified in alfalfa roots, indicating that Cd stress did not alter the fundamental classification of cell types (Fig. S6). However, Cd stress significantly altered the relative abundances of the cell types. Compared with those in the control group, the proportions of cortex (CK: 25.7%, Cd: 39.3%) and phloem (CK: 0.6%, Cd: 2.1%) cells significantly increased, suggesting that these cell types played greater roles in stress response or transport. Conversely, the proportions of meristematic (CK: 44.4%, Cd: 31.3%) and stem-niche (CK: 14.8%, Cd: 9.3%) cells markedly decreased, indicating suppression of cell proliferation. A modest increase was observed in the percentage of stele cells (CK: 5.2%, Cd: 9.1%), whereas the proportions of endodermis, epidermis, and xylem cells remained relatively stable (Fig. 4A, Table S3). These changes reflect the differential effects of Cd stress on distinct cell types.

Gene Ontology (GO) analysis revealed that both up- and downregulated differentially expressed genes (DEGs) were significantly enriched in cadmium-responsive processes, such as ‘response to Cd ions’ and ‘response to osmotic stress’, as well as in metabolic processes such as ‘lignin metabolic process’ and ‘phloem or xylem histogenesis’ (Figs 4D and E, S7A and B, Table S4, Table S5).

Consistent with the GO results, genes associated with heavy metal metabolism exhibited distinct tissue-specific expression patterns (Fig. 4F). For instance, MsHMA2 was specifically upregulated in stele cells, whereas MsHIPP3 was downregulated specifically in the xylem. Several genes showed broad but selective regulation; MsHMP20 was upregulated in all cell types except meristematic and stem-niche cells, whereas MsHMP52 and MsHIPP09 were downregulated in all but epidermal cells. In contrast, the expression of the metallothionein gene MsMT2A showed uniform downregulation across all cell types, and MsGSH1 exhibited significant expression changes across different cell types (Fig. 4G). To validate whether the specific expression results were associated with Cd stress, overexpression of MsGSH1 in yeast significantly enhanced Cd tolerance (Fig. 4H), confirming its role in stress response and validating the reliability of the transcriptomic findings.

### Cell differentiation trajectories of endodermis cells and phloem cells under Cd stress

**Figure 5:**
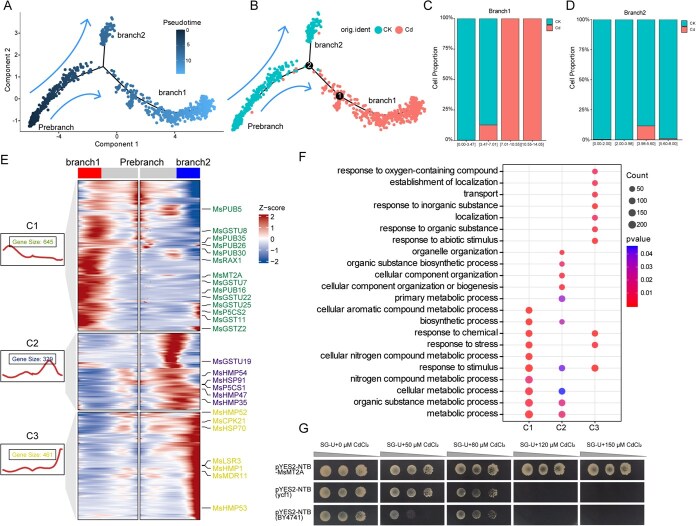
Pseudotemporal trajectory analysis reveals a Cd-induced shift in endodermal cell states. (A and B) Single-cell trajectory inference for endodermal cells, with pseudotime (A) and treatment condition (B), indicating a bifurcation point. (C and D) Proportional composition of control and Cd-treated cells along Branch 1 (C) and Branch 2 (D) of the trajectory. (E) Heatmap of gene expression dynamics along the pseudotime trajectory, grouped into three major coexpression clusters (left panel), with associated abiotic-stress-related genes highlighted (right panel). (F) GO biological process terms significantly enriched for genes within each cluster shown in (E). (G) Yeast spot assay confirming that *MsMT2A* expression increases survival under Cd stress.

Given the substantial number of DEGs identified in cells of the endodermis under Cd stress, we hypothesized that Cd exposure would likely significantly affect the developmental program of these cells. To test this hypothesis, we reconstructed the developmental trajectories of endodermis cells using single-cell transcriptomes from both control and Cd-stressed samples. Pseudotime analysis revealed that endodermis cells occupied five distinct states (Fig. S8). CytoTRACE analysis pinpointed State 1 as possessing the highest differentiation potential, designating it as the trajectory’s developmental origin. The inferred trajectory showed initial cells of the endodermis at the starting point and progressing toward maturity. Following a bifurcation event, the path diverged into two distinct branches (Branch 1 and Branch 2) (Fig. 5A and B), suggesting alternative physiological fates for maturing endodermis cells. Cd stress appeared to specifically affect Branch 1, leading to an accumulation of cells in the initial state and a depletion of mature cells in this branch. This pattern suggests that Cd stress may affect the normal developmental progression of endodermis cells specifically along this lineage. Quantifying cell proportions across four pseudotime-ordered stages within each branch further revealed a dynamic response: in Branch 1, the proportion of cells from Cd-stressed samples started at ~13% in early stages but increased progressively in later stages (Fig. 5C). Conversely, in Branch 2, the proportion of cells from Cd-stressed samples increased at the later stage but decreased gradually, reaching <12% by the later stage (Fig. 5D). Such branch-specific differences reflect the differentiation of distinct cellular fates within the endodermal transcriptome under Cd stress.

To identify key genes and pathways that might mediate the state transition of endodermis cells, we identified 1435 DEGs across the pseudotime order. These genes fell into three clusters with distinct gene expression patterns, representing a transcriptional rewiring program during the development of endodermis cells under Cd stress. There were 645 DEGs in Cluster 1 that were preferentially expressed in Branch 1. These genes were enriched in GO terms related to the responses to chemical, stress, stimulus, and biosynthetic processes. Some genes that responded to Cd stress, such as *MsPUB5*, *MsGSTU8*, *MsRAX1*, and *MsP5CS2*, were found in this cluster. Genes in Cluster 2 were highly expressed at the end of the prebranch and the start of Branch 2 (Fig. 5E, Table S6). GO analysis revealed that the genes in this cluster were significantly related to metabolic processes, organic substance metabolic processes, cellular component organization or biogenesis, and organelle organization (Fig. 5F). There were 461 DEGs in Cluster 3 that were expressed mainly at the end of Branch 2. These genes were enriched mainly in GO terms related to response to stimulus, response to abiotic stimulus, response to inorganic substance, and response to organic substance. To validate whether this branch-specific gene expression was linked to cellular fate and the Cd response, we selected MsMT2A, which encodes a metallothionein from Cluster 1, for functional validation. The results revealed that overexpression of MsMT2A in yeast significantly increased tolerance to Cd stress (Fig. 5G). Together, these findings demonstrate that Cd stress alters the normal differentiation program of endodermal cells and that branch-specific cell fate can, to some extent, predict the expression and function of associated genes such as MsMT2A.

**Figure 6:**
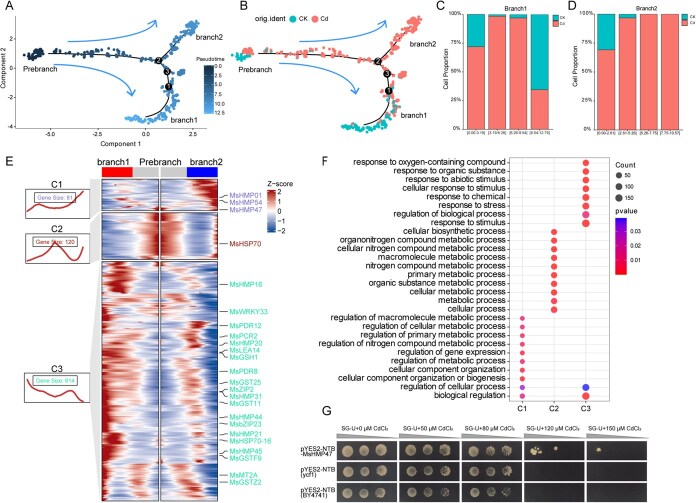
Pseudotemporal trajectory analysis reveals Cd-responsive pathways in phloem cells. (A and B) Reconstructed trajectory of phloem cells, colored by pseudotime (A) and treatment (B). (C and D) Distribution of control and Cd-treated cells along Branch 1 (C) and Branch 2 (D). (E) Heatmap displaying gene expression patterns along the trajectory, with clusters of heavy-metal-transport-related genes indicated. (F) GO enrichment analysis of the gene clusters defined in (E). (G) Yeast spot assay confirming that the expression of *MsHMA47* increases survival under Cd stress.

The observed significant increase in phloem cell abundance under Cd stress (CK: 0.6%, Cd: 2.1%) prompted us to investigate its impact on phloem cell development. To this end, we mapped the developmental trajectories of phloem cells using gene expression matrices from control and Cd-stressed plants. Pseudotime ordering resolved phloem cells into seven discrete states (Fig. S9). Assessment of differentiation potential via CytoTRACE identified State 6 as the most undifferentiated, establishing it as the developmental starting point. The trajectory began with these progenitor phloem cells and extended toward mature phloem identities. As in the endodermis, a bifurcation yielded two major branches (Branch 1 and Branch 2) (Fig. 6A and B), potentially representing divergent physiological endpoints for phloem maturation. Analysis of cell state distribution revealed that Cd stress led to a marked accumulation of progenitor-like cells (State 1) and a concomitant relative reduction in mature phloem cells, particularly within Branch 1. These observations were consistent with the hypothesis that Cd stress may disrupt the standard differentiation pathway of phloem cells in this branch. Stratifying both branches into four pseudotime segments and calculating the proportion of cells from Cd-stressed samples highlighted contrasting dynamics: along Branch 1, the fraction of stressed cells began at ~72% in early phases, increasing to 98% and then decreasing to 35% in later phases (Fig. 6C). In contrast, Branch 2 showed a gradual increase in the proportion of stressed cells, culminating at ~100% in later stages (Fig. 6D). This branch-specific temporal variation indicates that Cd stress induces significant yet heterogeneous shifts in differentiation pathways during phloem development.

To identify key genes and pathways that might mediate the state transition of endodermis cells, we identified 815 DEGs across the pseudotime order. These genes fell into three clusters with distinct gene expression patterns, representing a transcriptional rewiring program during the development of phloem cells under Cd stress (Fig. 6E). A total of 81 DEGs were found in Cluster 1 and were expressed mainly at the end of Branch 2. GO analysis revealed that these genes were significantly related to biological regulation, regulation of cellular processes, regulation of gene expression, and regulation of metabolic processes (Fig. 6F). Some heavy-metal-related genes were found in this cluster, such as *MsHMP01*, *MsHMP54*, and *MsHMP47*. The genes in Cluster 2 were expressed mainly at the start of the prebranch, which might be involved in the normal development of phloem cells. Cluster 3, which included 614 DEGs, was expressed mainly in the latter part of Branch 1 and the start of Branch 2. Some genes related to Cd stress were found in this cluster, for example, *MsPCR2*, *MsGST25*, *MsGSH1*, and *MsZIP2* (Table S7). GO analysis revealed that these genes were significantly enriched in the terms ‘response to oxygen-containing compounds’, ‘response to abiotic stimulus’, and ‘cellular response to stimulus’, indicating that Cd stress likely significantly influenced the development of phloem cells. To validate whether these genes were involved in the response to Cd stress, *MsHMP47*, which encodes a heavy-metal-stress-associated protein, was selected for functional verification (Fig. 6G). The results demonstrated that overexpression of *MsHMP47* in yeast significantly increased tolerance to Cd stress. In summary, our pseudotime analysis suggested that Cd stress may interfere with the differentiation process in both endodermis and phloem cells, although the precise underlying mechanisms require further investigation.

### Cell-type-specific chromatin accessibility and gene expression under Cd stress

**Figure 7:**
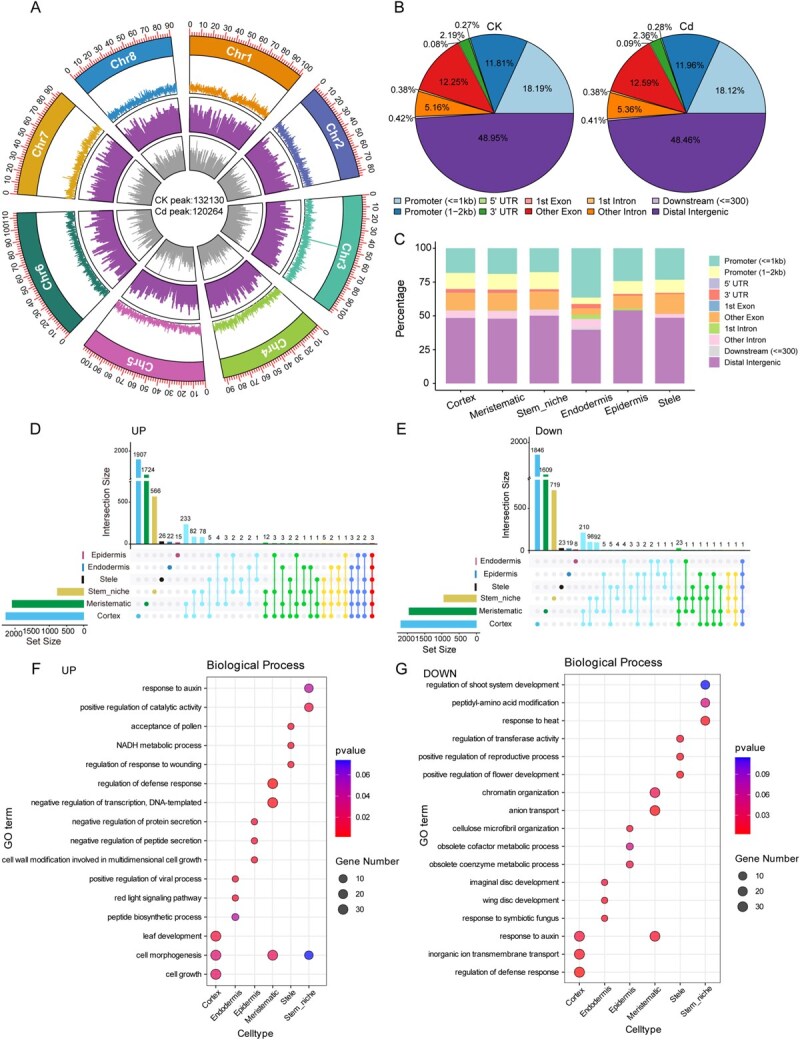
Cell-type-specific landscape of chromatin accessibility under Cd stress. (A) Genome-wide distribution of snATAC-seq peaks across chromosomes under control and Cd-stress conditions. (B) Genomic annotation of the identified peaks (e.g. promoter, intron, intergenic). (C) Distribution of peaks across genomic features for each cell type. (D, E) Bar plots showing the number of peaks with significantly increased (D) or decreased (E) chromatin accessibility in each cell type upon Cd stress. (F) GO analysis of the 4716 genes associated with upregulated peaks. (G) GO analysis of the 4688 genes associated with downregulated peaks.

On the basis of the above findings, we annotated the snATAC-seq data and identified eight distinct cell types, which was consistent with the snRNA-seq results (Fig. S10). Cortical cells accounted for the greatest proportion of the cells, followed by epidermal cells, whereas phloem and xylem cell types were relatively less abundant. Under normal conditions, a total of 132 310 peaks were identified, whereas under Cd stress, 120 264 peaks were detected (Fig. 7A, Table S8). Analysis of the number of peaks identified per cell type revealed that cortex and epidermis cells had the highest peak counts.

Further analysis of the distribution of the genomic peak revealed that under both normal and Cd-stress conditions, most peaks were located primarily in intergenic regions (CK: 48.95%, Cd: 48.46%), followed by promoter regions, particularly 1 kb upstream of the ATG start codon (CK: 18.19%, Cd: 18.12%). These findings indicated that Cd stress did not significantly alter the distribution patterns of the peaks (Fig. 7B). Additionally, a clear enrichment of peaks was observed near transcription start sites (TSSs) (Fig. S11).

To investigate whether Cd stress caused differences in chromatin accessibility across cell types, we performed differential peak analysis between normal and Cd-stress conditions for each cell type (Fig. 7C). In total, 11 087 differential peaks were identified, of which 5626 were upregulated (Fig. 7D) and 5461 were downregulated (Fig. 7E) under Cd stress. These differential peaks were distributed mainly in the cortex, meristematic, stem-niche, endodermis, epidermis, and stem cells. Genomic location analysis revealed that the majority (~50%) of the differential peaks were located in intergenic regions, followed by promoter regions. Notably, cortex and meristematic cells contributed the most cell-type-specific differential peaks, accounting for 64.5% of the upregulated and 63.3% of the downregulated differential peaks, respectively.

GO analysis of the 4716 genes associated with Cd-upregulated peaks revealed that different cell types were enriched in distinct biological processes (Fig. 7F). For instance, cortex cells were enriched primarily in cell growth and cell morphogenesis, whereas stem-niche cells were enriched in response to auxin and positive regulation of catalytic activity. Similarly, GO analysis of the 4688 genes linked to downregulated peaks revealed comparable functional enrichment (Fig. 7G), suggesting that different cell types respond to Cd stress by modulating the chromatin accessibility of genes involved in specific biological pathways.

**Figure 8:**
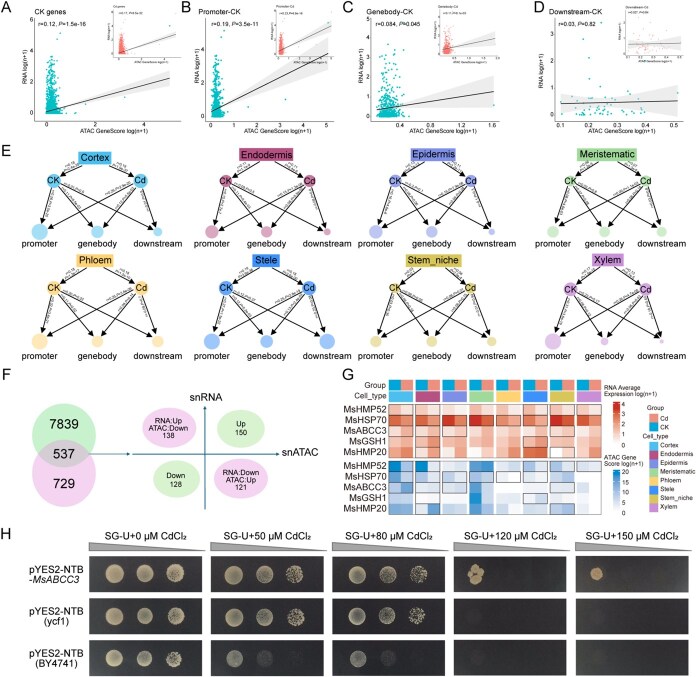
Integrative analysis of chromatin accessibility and gene expression. (A–D) Scatter plots showing the correlation between gene expression levels and chromatin accessibility in (A) all genomic regions, (B) promoter regions, (C) gene body regions, and (D) downstream regions. (E) Correlation analysis between chromatin accessibility and gene expression levels across eight cell types. (F) Venn diagram showing the overlap between genes with differential accessibility and differential expression. (G) Heatmap showing the expression patterns of five genes associated with heavy metal ion transport across eight cell types and two omics layers under normal and Cd-stress conditions. The border indicates genes whose expression trends were consistent across the two omics layers. (H) Yeast tolerance assay validating the function of *MsABCC3* under Cd stress.

We further explored the relationship between chromatin accessibility and gene expression from multiple perspectives. First, overall chromatin accessibility was weakly correlated with gene expression levels, with correlation coefficients of 0.12 under normal conditions and 0.17 under Cd stress (Fig. 8A). Second, when different genomic regions were examined, promoter accessibility exhibited the strongest correlation with gene expression, while the gene body and downstream regions showed weaker correlations (Fig. 8E). This trend slightly increased under Cd stress. Finally, cell-type-specific analysis indicated considerable variation in the correlation between chromatin accessibility and gene expression. For example, cortex, phloem, and stele cells were correlated above 0.15, whereas other cell types were correlated below 0.13 (Fig. 8B–D). Cd stress did not alter the overall positive correlation between accessibility and expression across cell types.

Furthermore, an integrated analysis linking differential chromatin accessibility with gene expression revealed that 74% (537 genes) of all the DEGs were associated with nearby differential ATAC peaks. Among these genes, 52% (278 genes) displayed consistent regulatory trends across the two omics layers—150 genes were concurrently upregulated in terms of both accessibility and expression, while 128 genes were co-downregulated (Fig. 8F). The remaining 259 genes showed opposing trends, further underscoring the multilayered complexity of transcriptional regulation. From the set of genes with consistent trends, *MsHMP52*, *MsHSP70*, *MsABCC3*, *MsGSH1*, and *MsHMP20* were selected for cell-type-specific heatmap analysis under different treatments, revealing robust coordinated signals across multiple cell types for all selected genes (Fig. 8G). For instance, the expression of MsABCC3 significantly increased chromatin accessibility and transcription, specifically in the cortex, endodermis, meristem, and stele-niche cells. To infer potential upstream regulators, we integrated TF expression profiles with the expression dynamics of their known or predicted target genes. This analysis revealed several candidate TF–target gene regulatory modules exhibiting cell-type-specific synergistic patterns. As a representative example, the *MsNAC*–*MsABCC3* module demonstrated concordant cell-type-specific chromatin accessibility and upregulation in both snRNA-seq and snATAC-seq datasets (Fig. S15). The functional relevance of this candidate module was further supported by heterologous assays, in which overexpression of *MsABCC3* in yeast significantly enhanced tolerance to Cd stress (Fig. 8H).

### Construction of a Cd-stress-responsive coexpression regulatory network in different cell types in alfalfa roots

**Figure 9:**
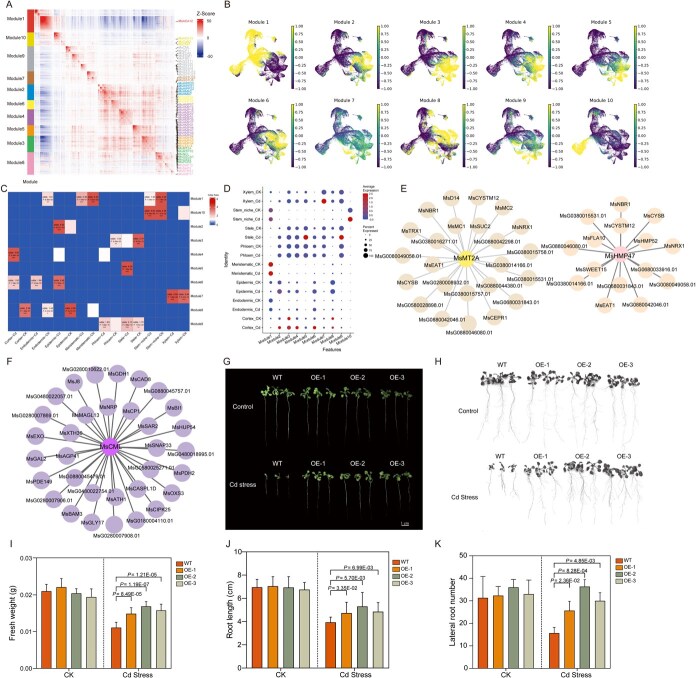
Coexpression regulatory network of Cd-stress responses in different cell types of alfalfa roots. (A) Hotspot analysis identifying gene modules with cell-type-specific expression patterns in response to Cd stress. (B) UMAP visualization of the 10 identified coexpression modules. (C) Correlation analysis between gene coexpression modules and different cell types under various conditions. (D) Dot plots of the cellular expression patterns of genes in 10 modules. (E, F) Gene coexpression networks centered on *MsMT2A* and *MsHMA47* (E) and on *MsCML* (F). Nodes are grouped according to module membership, and edges represent coexpression correlations. (G) Representative images of wild-type (WT) and the transgenic Arabidopsis plants overexpressing *MsCML* grown under control and Cd-stress conditions. (H) Scanned images of the root systems from (G). (I–K) Quantitative analysis of fresh weight (I), primary root length (J), and lateral root number (K) for the WT and transgenic lines under both conditions. The data are presented as the mean ± SE, and the asterisks indicate significant differences (Student’s *t* test; ^*^P < 0.05).

To construct a coexpression regulatory network of different cell types in alfalfa roots in response to Cd stress, a hotspot analysis was conducted. A total of 10 distinct coexpression modules, comprising 3455 genes, were identified (Fig. 9A and Fig. S12, Table S9). The number of genes in each coexpression module varied significantly, with the smallest being Module 6, containing only 192 genes, and the largest being Module 9, containing 526 genes (Fig. 9B and Fig. S13). Among these coexpressed genes, we identified several genes known to be related to Cd stress, such as *MsACA12*, *MsACA13*, *MsCaN2*, and *MsCML* (associated with the Ca^2+^ signaling pathway); *MsGST11*, *MsGST25*, *MsGSTF9*, *MsGSTT2*, and *MsGSTU19* (related to glutathione S-transferase); and *MsHIPP20*, *MsHIPP21*, *MsHIPP22*, *MsHMA2*, *MsHMA47*, *MsMT2A*, and *MsMTP10* (heavy-metal-related genes).

The expression patterns of genes within these modules were subsequently characterized. Different coexpression modules exhibited specific cell-specific expression patterns. For example, genes in Module 1 and Module 10 were expressed primarily in meristematic and stem-niche cells, genes in Module 2 were expressed mainly in epidermis cells, genes in Modules 4 and 6 were expressed predominantly in cortex cells, genes in Module 7 were expressed primarily in xylem cells, and genes in Module 9 were expressed mainly in phloem and stele cells. Correlation analysis between the expression abundance of coexpressed gene modules and cell types indicated that these modules were significantly associated with specific cell types under Cd stress (Fig. 9C). Module 1 was related primarily to meristematic cell types under Cd stress (odds ratio: 2.36, *P* = 1.54e-07), Module 2 was associated mainly with epidermis cell types (odds ratio: 4.00, *P* = 6.67e-123), Module 4 was linked predominantly to cortex cell types (odds ratio: 3.90, *P* = 9.48e-24), and Module 9 was significantly correlated with phloem and stele cell types. Additionally, Cd stress significantly affected the expression abundance of these genes. The expression abundance of genes in Module 10 significantly increased in stem-niche cells under Cd stress, and the expression of genes in Modules 5 and 9 significantly increased in stele cells. The expression of genes in Module 7 significantly increased in the xylem cells, and similarly, the expression of genes in Module 4 significantly increased in the cortex cells (Fig. 9D). GO analysis revealed that these genes in different modules were significantly involved in biological processes. For example, genes in Module 4 were significantly related to ‘response to toxic substance’, and genes in Module 9 were significantly related to ‘metal ion transport’, ‘metal ion homeostasis’, and ‘cellular ion homeostasis’ (Fig. S13).

Coexpression regulatory networks for *MsMT2A* and *MsHMA47*, two genes reported to significantly increase Cd tolerance in yeast, were constructed. *MsMT2A* and *MsHMA47* belonged to module 9, where genes showed increased expression abundance primarily in stele cells under Cd stress. The coexpression network of *MsMT2A* contained 24 DEGs, including *MsEAT1*, which is related to ethylene, and *MsSUC2*, which is involved in plant sucrose metabolism (Fig. 9E, Table S10). The coexpression network of *MsHMA47* contained 15 DEGs, including *MsHMA52*, which is associated with heavy metal stress, and *MsSWEET15*, which is related to plant sucrose transport (Table S11).

Furthermore, we identified 33 DEGs in Module 4, including *MsCIPK25* and *MsCP1*, involved in plant Ca^2+^ signaling; *MsOXS3*, related to oxidative stress; and *MsATH1*, an ATP-binding cassette family gene associated with Cd stress. Notably, all these genes were coexpressed with *MsCML* (Fig. 9F, Table S12). Therefore, we hypothesized that *MsCML* might participate in the plant response to Cd stress. To validate this hypothesis, we overexpressed *MsCML* in Arabidopsis and obtained three independent overexpression transgenic lines. The results revealed that the growth status of the *MsCML*-overexpressing transgenic lines under Cd stress was greater than that of the wild-type plants, with significantly greater root length, lateral root number, and fresh weight, indicating that overexpression of *MsCML* significantly increased Cd tolerance in the transgenic lines (Fig. 9G–K).

## Discussion

Plants exhibit cellular heterogeneity in response to heavy metal stress, with different cell types playing distinct roles in coordinating overall tolerance and accumulation mechanisms [53]. The rapid advancements in snRNA-seq technology provide an unprecedented opportunity to decipher this cellular heterogeneity and the underlying molecular regulatory networks at single-cell resolution [54]. This technique directly sequences nuclei, bypassing the need for protoplast isolation, thus effectively avoiding potential artifacts introduced by enzymatic digestion and enabling the in-depth analysis of plant tissues with rigid cell walls [55]. In this study, we performed snRNA-seq and snATAC-seq analyses of nuclei isolated the from root cells of alfalfa under both control and Cd-stress conditions, providing the first systematic characterization of the heterogeneous responses to Cd stress at single-cell resolution.

In this study, metabolomic analysis was first performed on alfalfa roots from the control and Cd-stress treatment groups, and a total of 918 metabolites whose metabolic profiles significantly differed between the two groups were identified. Enrichment analysis of the DAMs revealed that the most significantly enriched pathways included ‘galactose metabolism’, ‘ABC transporters’, and ‘phenylpropanoid biosynthesis’. These results indicate that Cd stress induces extensive remodeling of root metabolites, involving important physiological processes such as energy metabolism, transmembrane transport, and the synthesis of secondary metabolites. This marked metabolic response demonstrated that the experimental treatment was effective and that the samples maintained active metabolic and stress-responsive states under stress, with overall physiological functions not suffering catastrophic disruption. Therefore, conducting simultaneous snRNA-seq and snATAC-seq analyses at this critical stage of clear metabolic remodeling is fully justified. The profound changes in metabolites necessarily arise from coordinated alterations in gene expression and epigenetic regulation. Multiomics integration can systematically reveal the complete regulatory cascade from chromatin accessibility dynamics to transcriptional activation and, ultimately, to metabolic phenotypes, thereby enhancing the explanatory power and causal persuasiveness of the findings at the mechanistic level.

Through snRNA-seq analysis, we successfully identified and annotated the major cell types in the roots, including cortex cells, endodermis cells, epidermis cells, meristematic cells, stem-niche cells, phloem cells, stele cells, and xylem cells. While Cd stress did not alter the fundamental cell type classification, it significantly reshaped the proportional abundance of these genes. For instance, the proportions of meristematic cells (CK: 44.4%, Cd: 31.3%) and stem-niche cells (CK: 14.8%, Cd: 9.3%) decreased significantly, whereas those of cortex cells (CK: 25.7%, Cd: 39.3%) and phloem cells (CK: 0.6%, Cd: 2.1%) increased markedly. These findings indicated an adaptive reconfiguration of the root cell population in response to Cd stress, shifting from a ‘growth mode’ to a ‘defense mode’. Previous studies have reported similar findings. Cd stress does not affect the cell types within tissues but rather causes shifts in their proportions [37]. This cellular reallocation represents a core strategy for sacrificing growth to reinforce defense mechanisms against stress. These findings provide direct cellular evidence for understanding how heavy metal stress impairs the critical symbiotic nitrogen fixation function in legumes.

Through single-cell resolution analysis, we revealed that different heavy metal ATPase (HMA) family genes exhibit highly distinct expression patterns across identified cell types, suggesting their roles in mediating compartmentalized Cd detoxification and transport strategies. Specifically, the specific upregulation of *MsHMA2* in stele cells indicates its potential role in loading Cd^2+^ into the xylem cells, thereby facilitating Cd translocation from roots to shoots or sequestration. In contrast, the downregulation of *MsHIPP3* in xylem cells may represent an adaptive regulatory mechanism to limit Cd migration to shoots. Unlike these cell-type-specific patterns, *MsMT2A*, which encodes a metallothionein, was strongly induced across all examined cell types, confirming that metal chelation is a universal and fundamental cellular defense mechanism—a finding consistent with previous reports [37]. Furthermore, the upregulated expression of *MsGSH1* in phloem, endodermis, and stele cells was further functionally validated: heterologous overexpression of *MsGSH1* in yeast significantly enhanced Cd tolerance, demonstrating that this gene positively regulates plant Cd detoxification by boosting glutathione biosynthesis. These findings are consistent with previous conclusions regarding the cell-type-specific expression of cadmium-stress-responsive genes [37]. At the mechanistic level, our results also connect with established pathways. For instance, in Arabidopsis, GSH1-mediated glutathione synthesis is not only a core process in the cadmium-stress response but also acts as a downstream effector of the SNAT-mediated melatonin signaling pathway [56]. Therefore, this study deepens the understanding of the fine-tuned regulatory network underlying plant cadmium-stress responses from a cell-type-specific perspective. It should be noted that the functional validation in this study was primarily conducted in a heterologous yeast system, and the general applicability of these conclusions in alfalfa itself requires further verification.

To gain in-depth insights into the dynamic impact of Cd stress on cellular developmental processes, we employed pseudotime trajectory analysis, a computational method that orders cells along a pseudotemporal trajectory on the basis of continuous changes in their transcriptomes, thereby reconstructing dynamic gene expression programs during biological processes. Pseudotime analysis has been widely used to study cell differentiation [57, 58], immune responses [59, 60], disease progression [61, 62], and many other biological systems with temporal dynamics. Our analysis revealed that Cd stress disrupted the normal developmental trajectories of endodermal and phloem cells in alfalfa roots. In endodermal cells, Cd specifically blocked the cell maturation process along Branch 1 and activated a stress-responsive program comprising 645 genes, including *MsMT2A*. Yeast overexpression assays confirmed that *MsMT2A* significantly increased Cd tolerance, supporting its role as a key factor in the adaptive defense mechanism of this branch. In *A. thaliana, MT2A* is known to mediate ROS homeostasis during oxidative stress and participate in senescence and the copper response [63], further supporting its conserved function in heavy metal detoxification. In phloem cells, Cd stress induced distinct branch-specific responses. A core cadmium-stress response module consisting of 614 highly expressed genes, including *MsHMP47* and *MsGSH1*, was identified in the mid-to-late stage of Branch 1. Functional validation via yeast overexpression demonstrated the detoxification role of *MsHMP47* under Cd stress, which is consistent with reports that most HMP gene family members in rice and Arabidopsis respond to heavy metal stress [64]. In summary, Cd stress drives cell fate reprogramming through key genes such as *MsMT2A* and *MsHMP47*, redirecting developmental trajectories toward branch paths characterized by the activation of specific detoxification and defense programs. These findings indicate that Cd stress influences cell fate decisions, channeling the developmental trajectory toward branching paths characterized by the activation of specific detoxification and defense programs. This discovery reveals the strategy by which plants orchestrate cell fate at the single-cell level to cope with environmental stress.

Chromatin accessibility is a central mechanism in the regulation of gene expression, as it determines whether regulatory factors can bind to *cis*-regulatory elements on DNA, thereby influencing gene expression [65]. This regulatory relationship exhibits high cell-type specificity and environmental context dependency. snATAC-seq provides a powerful tool for elucidating the relationship between chromatin accessibility and gene expression across different cell types under varying environmental conditions. In this study, we employed snATAC-seq to elucidate heterogeneous changes in chromatin accessibility among different cell types in the roots of alfalfa under Cd stress. The number of differentially accessible peaks varied significantly across cell types: certain cell types (such as cortical cells) exhibited many differential peaks, suggesting extensive chromatin remodeling in these cells in response to Cd stress. In contrast, some cell types showed no significant differential peaks, possibly indicating either insensitivity of their chromatin structure to Cd stress or a rapid response mechanism at other levels (such as post-transcriptional regulation). This cell-type-specific epigenetic response highlights the importance of investigating abiotic stress mechanisms at single-cell resolution.

Prior studies have established a positive correlation between chromatin accessibility and gene expression levels [66, 67], which is also observed in our dataset (*R* = 0.12, *P* = 1.5e-16). However, the strength and nature of this association vary across cell types. For instance, the strongest correlation was detected in the stele cells (*R* = 0.19, *P* = 3.6e-36), whereas a weaker association was detected in the stem-niche cells (*R* = 0.06, *P* = 2.5e-4). Analysis of differential peaks and DEGs revealed that approximately half of the genes exhibited discordant regulatory patterns between the two omics layers. We speculate that these discrepancies may arise for the following reasons. First, an open chromatin region may provide a binding site not only for TFs that activate gene expression but also for those that repress it. Second, gene expression levels are influenced not only by transcriptional regulation but also by posttranscriptional mechanisms, such as low translation efficiency or degradation mediated by microRNAs or RNA-binding proteins [68]. Through integrative analysis of the relationship between differential chromatin accessibility and gene expression, we found that 52% of genes (278 genes) exhibited consistent regulatory trends, while the remaining genes showed opposing trends, further revealing the complexity of transcriptional regulation. From the genes with consistent trends, we selected MsHMP52, MsHSP70, MsABCC3, MsGSH1, and MsHMP20 for cell-type-specific heatmap analysis under different treatment conditions and found that the selected genes all exhibited strong synergistic signals across different cell types. To further investigate the upstream regulatory mechanisms, we focused on MsABCC3 as a representative case and integrated TF expression profiles with the expression dynamics of its known or predicted target genes to infer its upstream TFs. Multiple candidate TF–target gene regulatory modules were identified, all displaying cell-type-specific synergistic patterns. Among these, the MsNAC-MsABCC3 module exhibited consistency in cell-type-specific chromatin accessibility and upregulated expression in both snRNA-seq and snATAC-seq datasets. Further heterologous validation experiments demonstrated that overexpression of *MsABCC3* in yeast significantly enhanced cadmium-stress tolerance, confirming the functional relevance of this candidate module.

Weighted gene coexpression network analysis (WGCNA) can identify covarying gene modules and elucidate their biological functions [69, 70]. This method clusters coordinately regulated genes into modules by analyzing gene expression correlations; these modules typically correspond to specific biological processes or regulatory networks and have been successfully applied to identify key genes involved in crop stress responses [31]. On the basis of the snRNA-seq data from this study, we constructed a coexpression network and identified 10 gene modules that were significantly associated with specific cell types. The analysis revealed distinct module activity patterns among different cell types. Notably, endodermal cells underwent a marked state transition. Under control conditions, endodermal cells were significantly associated with Module 8 and Module 1, whereas under Cd stress, their association shifted significantly to Module 6. GO enrichment analysis revealed that Module 8 and Module 1 were enriched primarily in basic biosynthetic processes, whereas Module 6 was significantly enriched in phenylpropanoid metabolism, flavonoid biosynthesis, and sulfur compound metabolic processes. This module transition trajectory aligns closely with the adaptive reprogramming of endodermal cells under stress, suggesting a shift from maintaining basic metabolism toward activating specific secondary metabolic detoxification pathways—a short-term defense strategy potentially achieved at the cost of temporarily halting normal developmental progression. Similarly, phloem cells also exhibited adaptive functional changes. This cell type was primarily associated with Module 3 and Module 9, both before and after stress, with the activity of Module 9 changing most significantly under stress. Functional enrichment analysis suggests that gene expression in phloem-related cells shifted from normal protein and hormone transport toward pathways related to metal ion transport and homeostasis, which may reflect an adjustment in transported substances to adapt to Cd stress.

The correlation analysis between modules and cell types mutually validated the above findings and helped us focus on key regulatory genes. Notably, *MsCML* emerged as a highly connected hub gene within a coexpression module strongly associated with Cd stress. On this basis, we conducted a preliminary functional validation using a heterologous Arabidopsis transformation system. Although differences exist between the heterologous system and the native alfalfa environment, the functional assay results demonstrated that overexpression of MsCML significantly enhanced Cd tolerance in Arabidopsis, supporting its positive role in the Cd-stress response and establishing it as a candidate gene for further investigation.

In summary, by integrating snRNA-seq with genome-wide WGCNA, this study elucidates cell-type-specific transcriptional regulatory networks in alfalfa roots under Cd stress. We found that distinct cell types respond to Cd toxicity by regulating specific gene sets, including *MsMT2A*, *MsGSH1*, *MsHMP47*, *MsABCC3*, and *MsCML*. Heterologous overexpression of these genes in yeast or Arabidopsis enhanced Cd tolerance, functionally corroborating their roles in stress responses. This study provides novel insights into heavy metal tolerance mechanisms in legumes at single-cell resolution. More importantly, by integrating chromatin accessibility (snATAC-seq) data with transcriptomic profiles, we moved beyond the mere identification of DEGs to precisely pinpoint high-confidence regulatory hubs (e.g. the MsNAC-MsABCC3 module and the hub gene MsCML). These hubs were substantiated by multiomics evidence, including coordinated cell-type-specific expression and chromatin accessibility. Consequently, this study establishes a high-confidence candidate gene repository for alfalfa molecular breeding. These regulatory nodes, validated by both single-cell resolution and epigenetic mapping, provide foundational resources for developing stress-tolerant forage germplasm and for constructing synthetic biological systems with enhanced tolerance.

## Materials and methods

### Plant materials and growth conditions

The alfalfa (*Medicago sativa*) cultivar ‘Zhongmu No. 4’ was used in this study. The seeds preserved at the laboratory of the Institute of Animal Sciences, Chinese Academy of Agricultural Sciences, were evenly placed on moist filter paper and stratified at 4°C in the dark for 3 days to promote germination. The germinated seeds were then transferred to a greenhouse and grown for 3 weeks under the following conditions: a 16/8-h light/dark cycle, 75% relative humidity, and a day/night temperature of 20/24°C.

### Cd-stress treatment and sample collection

Three-week-old alfalfa seedlings were subjected to Cd-stress treatment. They were exposed to Hoagland’s nutrient solution supplemented with 45, 90, or 135 μM CdCl₂, constituting three independent treatment groups. A control group (CK) was grown in Hoagland’s nutrient solution without CdCl₂. After 5 days of treatment, the root samples were collected, thoroughly rinsed five times with 5 mM CaCl₂ solution to remove surface residues, and then immediately frozen in liquid nitrogen. All the samples were stored at −80°C for subsequent snRNA-seq and snATAC-seq analyses. Three independent biological replicates were prepared for both the control group and each treatment group.

### Metabolomics analysis

Metabolite profiling of cells treated with 90 μM CdCl₂ was performed using liquid chromatography–tandem mass spectrometry (LC–MS/MS). The analytical platform consisted of an Exion LC UPLC system coupled with a QTRAP 6500+ mass spectrometer. Data acquisition was conducted in multiple reaction monitoring (MRM) mode. Raw mass spectrometry data were processed using SCIEX OS software, with key parameters set as follows: minimum peak height of 500, signal-to-noise ratio of 5, and smoothing points of 1, to perform peak integration, alignment, and correction, thereby obtaining qualitative and quantitative results for the metabolites. Metabolite identification was carried out by Beijing Novogene Bioinformatics Technology Co., Ltd. DAMs were identified according to multiple criteria. The variable importance in projection (VIP) values from the first principal component of the partial least squares discriminant analysis (PLS-DA) model were calculated using metaX software [71]. These data were combined with *P* values from Student’s *t* test and fold change (FC) values. The criteria for identifying DAMs were set as follows: VIP > 1, *P* < 0.05, and FC ≥ 1.5 or FC ≤ 0.667. Categorical statistics of the identified metabolites were performed using GraphPad Prism software. KEGG pathway enrichment analysis of the DAMs was conducted using the OmicShare online platform, with a *P* value < 0.05 considered to indicate statistically significant enrichment.

### Nucleus isolation

The nuclear isolation procedure was modified from the product information sheet of the CelLytic™ PN Isolation/Extraction Kit (Sigma). The root tips were chopped and mixed in 1× NIBTA buffer (1× NIB, 1 mM dithiothreitol, 1× ProtectRNA RNase inhibitor, 1× cOmplete, EDTA-free Protease Inhibitor Cocktail, and 0.3% Triton X-100) on prechilled plates, and the homogenates were transferred to 15-ml tubes. The mixture was shaken gently on ice for 5 min, after which it was passed through a 40-μm filter. The filtrate was collected into a new 15-ml tube and centrifuged at 1260 g for 10 min, after which the supernatant was discarded, and the pelleted nuclei were resuspended in 4 ml of 1× NIBTA buffer. The nuclear resuspension was added to a new 15-ml tube containing 80% Percoll solution (4 ml Percoll and 1 ml NIBTA buffer). After centrifugation at 800× g for 30 min, the nuclei were located in the 1× NIBTA buffer and Percoll interface bands. The nuclei were gently collected into new 15 ml tubes, 10 ml of 1× NIBTA buffer was added, and the samples were centrifuged at 1260 g for 5 min. Finally, the nuclei were washed twice in 1× NIBTA buffer and resuspended in phosphate-buffered saline (PBS) containing 0.04% bovine serum albumin (BSA) to a final concentration of 3000 nuclei/μl (for snRNA-seq) or in PBS containing 1% BSA to a final concentration of 6000 nuclei/μl (for snATAC-seq).

### Single-nucleus RNA library construction and sequencing

The obtained single-nucleus suspensions were used for snRNA-seq library preparation according to the DNBelab C Series Single-Cell Library Prep Set (MGI, 1000021082) as previously described [72]. The concentration of the DNA library was measured by a Qubit ssDNA Assay Kit (Thermo Fisher Scientific). Libraries were sequenced by DNBSEQ-T7. The read length was as follows: Read 1 was 30 bp, including the 10-bp cell Barcode 1, 10-bp cell Barcode 2, and 10-bp UMI, and Read 2 was 100 bp for the transcript and 10 bp for the sample index.

### Single-nucleus ATAC library construction and sequencing

The snATAC-seq libraries were prepared as previously described [73] using the DNBelab C Series Single-Cell ATAC Library Prep Set (MGI, 1000021878). Briefly, nuclei were extracted from tissue following the same protocol as described above. Indexed libraries were generated according to the manufacturer’s instructions, and concentrations were determined using the Qubit ssDNA Assay Kit (Thermo Fisher Scientific). The libraries were sequenced on a DNBSEQ-T7 platform with the following sequencing strategy: 70 bp read length for Read 1, including 10 bp for cell Barcode 1, 10 bp for cell Barcode 2, and 50 bp for open chromatin; Read 2 was 50 bp for open chromatin and 10 bp for sample indexing.

### Processing of the snRNA-seq data

The raw sequencing data were aligned to the *Medicago sativa* genome (https://figshare.com/articles/dataset/Medicago_sativa_genome_and_annotation_files/12623960). Initial processing was performed using the DNBC4tools [74] (version 2.1.3) pipeline for gene counting. The resulting raw count matrix was further analyzed in R using the Seurat package [75] (version 4.3.0.1). Gene filtering was applied, and genes expressed in at least three cells were retained (Seurat parameter: min.cells = 3). Cell filtering was conducted by retaining cells with a minimum of 200 expressed genes and a maximum of 8000 expressed genes (min.features = 200, nfeature_RNA < 8000). Background noise was removed using SoupX [76] (version 1.6.2) with default settings apart from the contamination fraction. Doublets were identified and removed using DoubletFinder [77] (version 2.0.3). DoubletFinder first averages the transcriptional profile of randomly chosen cell pairs to create pseudodoublets and then predicts doublets according to each real cell’s similarity in gene expression to the pseudodoublets. Doublet removal was performed with the default parameters of DoubletFinder. Batch effects were corrected using canonical correlation analysis (CCA). Data normalization was performed using the LogNormalize tool (NormalizeData function in Seurat) with a scale factor of 10 000. Variable genes were identified using the vst method (FindVariableFeatures function in Seurat), and the top 2000 most variable genes were selected. The data were then scaled using the ScaleData function. PCA was performed on the 2000 variable genes, and dimensionality reduction was performed, with visualization using UMAP and t-SNE. Marker genes for each cluster were identified using the Findallmarkers function in Seurat with the Wilcoxon rank-sum test to assess differential expression. To account for multiple testing across all genes, the Benjamini-Hochberg procedure was applied to control the false discovery rate (FDR). Cluster-enriched genes were defined using a threshold of min.logfc = 0.25 and padj <0.05. Cell types were annotated on the basis of known cell-type-specific marker genes [78], as referenced in Table S1.

### SnATAC-seq data processing and cell annotation

Single-cell ATAC-seq data were processed using a comprehensive pipeline. The raw FASTQ files were processed with dnbc4tools (version 2.1.3) for barcode demultiplexing and quality control, followed by alignment with the *Medicago sativa* reference genome. Quality control was performed by retaining cells with more than 1000 unique nuclear fragments and TSS enrichment scores >1.5. Potential doublets were removed using the default doublet filtering functions (addDoubletScores) in ArchR [79] (version 1.0.1). The Harmony tool [80] integrated into ArchR was used to correct for the batch effect. Clustering analysis was performed using ArchR by first identifying a robust set of peak regions followed by iterative latent semantic indexing (LSI) clustering. In brief, 500-bp tiles were created across the genome, and whether each cell was accessible within each tile was determined. Next, the LSI dimensionality was reduced on these tiles with the addIterativeLSI function in ArchR. Seurat clustering (FindClusters) was performed on the LSI dimensions at a resolution of 0.6. Anchors between the snATAC-seq and snRNA-seq datasets were identified and used to transfer cell type labels identified from the snRNA-seq data. Data were coembedded using the TransferData function of Seurat [72].

### Single-cell differential expression and enrichment analysis

DEGs were identified between sample groups using the FindMarkers function with stringent thresholds. The upregulated genes had *P*_adj_ <0.05 and log2FC ≥ 0.25, whereas the downregulated genes had *P*_adj_ <0.05 and log2FC ≤ −0.25 [45]. The UpSetR R package (version 1.4.0) was used to visualize the complex relationships among DEG sets across different comparisons, highlighting intersecting and unique gene expression patterns in an intuitive matrix layout [81]. The functional characterization of the DEGs was performed on the basis of GO biological process annotations derived from the reference genome. Pathway enrichment analysis was performed using the KEGG database. Both GO and KEGG analyses were performed using the clusterProfiler package [82].

### snATAC-seq peak detection

Peak calling was performed for each cell type within each group. For peak identification and reproducibility assessment, we utilized the ArchR pipeline, specifically the addReproduciblePeakSet method, which enables the detection of regions with consistent chromatin accessibility across multiple samples or conditions. Differential peaks of sample groups were identified by the getMarkerFeatures function with default parameters [73].

### Pseudotime analysis and differential gene expression

To analyze differentiation trajectories and determine cell fate, pseudotime analysis was conducted using the Monocle2 [83] (version 2.10.0) and CytoTRACE R packages [84]. A subset of raw data containing the target clusters was analyzed using Monocle2. Variable genes were identified with the dispersionTable function on the basis of average expression levels. Dimensionality reduction was performed with the DDRTree method (max_components = 2), and cell ordering was conducted with the orderCells function. The trajectory was visualized with the plot_cell_trajectory function, and the root state was specified by rerunning orderCells with the root_state argument. Dynamically expressed genes along pseudotime were clustered and visualized with the plot_pseudotime_heatmap function. Branch-dependent genes were identified using the differentialGeneTest function at selected branch points and visualized with the plot_genes_branched_heatmap function. Each gene cluster was subjected to KEGG and GO enrichment analyses to explore the regulatory networks and functional pathways involved in differentiation.

### Transcription factor regulatory network analysis

TF regulatory network analysis was performed using the pySCENIC pipeline (v0.11.2) [85]. Processed snRNA-seq data with cell type annotations were converted into loom files and used as inputs for SCENIC analysis. Gene regulatory networks were inferred using GRNBoost to identify putative TF–target relationships, followed by cis-regulatory motif enrichment analysis with RcisTarget to retain direct regulatory interactions. Regulon activity was quantified at the single-cell level using AUCell, enabling the identification of cell-type-specific TF activity patterns. Visualization of TF expression and regulon activity was performed using igraph.

### Hotspot analysis and gene coexpression network analysis

To identify functionally related gene modules, a gene coexpression network analysis was performed. First, highly variable genes (HVGs) were identified on the basis of the dispersion of their expression across all cells, and the top 6000 HVGs were retained. Subsequently, gene–gene similarity networks were constructed using the Hotspot [69] algorithm (version 1.1.1) by creating an unweighted k-nearest neighbor (KNN) graph with 30 neighbors per gene (n_neighbors = 30, weighted_graph = false). Coexpression modules were identified by applying stringent criteria, with each module being required to contain at least 50 genes (min_gene_threshold = 50) and retaining only core genes with significant coexpression patterns (core_only = True) at an FDR threshold of 0.05 (fdr_threshold = 0.05). The identified modules were subjected to functional enrichment analysis to explore the biological pathways and processes associated with each coexpressed gene set.

### Yeast transformation and phenotypic verification

On the basis of small RNA sequencing data, we identified four genes that were significantly downregulated in at least one cell type under Cd stress (log₂FC ≤ −0.25, corrected *P*_value < 0.05). The recombinant plasmids pYES2-NTB-MsGSH1, pYES2-NTB-MsABCC3, pYES2-NTB-MsMT2A, and pYES2-NTB-MsHMP47, along with the empty vector controls, were individually introduced into the yeast competent strain ycf1. The detailed transformation procedure (involving the LiAc/PEG method preparation and heat shock program) is provided in Supplementary Method S1. Transformants were spread onto SD-Ura dropout selection plates and incubated at 30°C for 3–5 days to screen for positive clones.

To verify the Cd tolerance of the transformants, single colonies previously confirmed by PCR were suspended, serially diluted, and spotted onto SG-Ura solid plates (with galactose as the carbon source) containing varying concentrations of CdCl₂ (0 to 200 μM). The plates were incubated at 30°C, and growth was observed and documented after 3–7 days of cultivation.

### Development of transgenic Arabidopsis overexpression lines

To investigate the function of MsCML70 in the Cd-stress response, its full-length cDNA was cloned and inserted into the PBWA(V) BS vector containing the 35S promoter. Following an established method [86], Arabidopsis was transformed via the Agrobacterium-mediated floral dip method to obtain transgenic plants. T0 generation seeds were screened on one-half MS medium supplemented with 50 mg/L kanamycin to select positive seedlings. The plants were propagated to obtain homozygous T3 generation lines, which were subsequently used for functional analyses.

### qRT–PCR analysis of transgenic plants

The seeds of the homozygous T3 transgenic Arabidopsis lines were surface-sterilized, stratified at 4°C, and then sown on one-half MS medium supplemented with 60 mg/L kanamycin. The plants were grown in a growth chamber (24/20°C, 16/8 h light/dark cycle) for 10–14 days before being transplanted into soil and cultivated for an additional 3–4 weeks. Total RNA was extracted from leaf tissues using the TRIzol method. qRT–PCR was performed using gene-specific primers for MsCML70 and the reference gene AtActin (the reagents and instruments used are detailed in Supplementary Method S2). Each reaction was performed with three technical replicates. Relative expression levels were calculated using the 2–ΔΔCT method, confirming the successful overexpression of MsCML70 in the transgenic lines (Fig. S14). Three homozygous lines with high expression levels were selected for subsequent phenotypic analysis.

### Phenotypic analysis of transgenic Arabidopsis seedlings under Cd stress

Seeds of both wild-type and transgenic Arabidopsis plants were surface-sterilized, stratified, and sown on one-half MS solid media. After uniform germination, seedlings were transferred to fresh one-half MS media supplemented with either 0 or 75 μM CdCl₂ for vertical cultivation. After 14 days of growth, phenotypic parameters, including primary root length, fresh weight, and lateral root number, were measured for the seedlings of each line. The experiment employed a completely randomized design. Each treatment consisted of at least 12 seedlings (constituting one biological replicate), and the entire experiment was independently repeated three times. The data are presented as the mean ± standard deviation. Statistical analysis involving one-way analysis of variance followed by Duncan’s new multiple-range test was performed using SPSS software.

## Supplementary Material

Web_Material_uhag117

## Data Availability

All the data generated or analyzed in this study are fully provided in the supplementary attachments and are available in structured formats (e.g. xlsx). No additional access requests are required for verification, reproduction, or further analysis. For inquiries regarding the data, please contact the corresponding author as indicated in the document.
