# Supplementary material for: Single-nucleus RNA-seq and ATAC-seq analyses provide molecular insights into cadmium-stress response in alfalfa roots
Source: Hortic Res. 2026 Apr 6;13(8):uhag117. doi: 10.1093/hr/uhag117 (PMC13392044; doi:10.1093/hr/uhag117)
Supplement: Web_Material_uhag117 [file web_material_uhag117.zip › supplementary materials .docx]

**Supplementary data for**

**Single-nucleus RNA-seq and ATAC-seq analyses provide molecular insights into the cadmium stress response in alfalfa roots**

Yuqi Zhang^1+^, Hao Liu^1+^, Ming Xu1^+^ , Mengjia Xie^2+^, Shuhan Deng^2+^, Xinyue Ma^1^, Li Zhao^1^, Fei He^1^, Mingna Li^1^, Ruicai Long^1^, Xue Wang^1^, Junmei Kang^1^, Qingchuan Yang^1^, Lin Chen*^1^

1.Institute of Animal Science, Chinese Academy of Agricultural Sciences, Beijing 100193, China.

2.Glbizzia Biosciences Co., Ltd., Beijing 102609, China.

^+^ These authors contributed equally to this work

* Corresponding author: Lin Chen, email: [chenlin@caas.cn](mailto:chenlin@caas.cn)

**The authors declare no conflict of interest.**

**
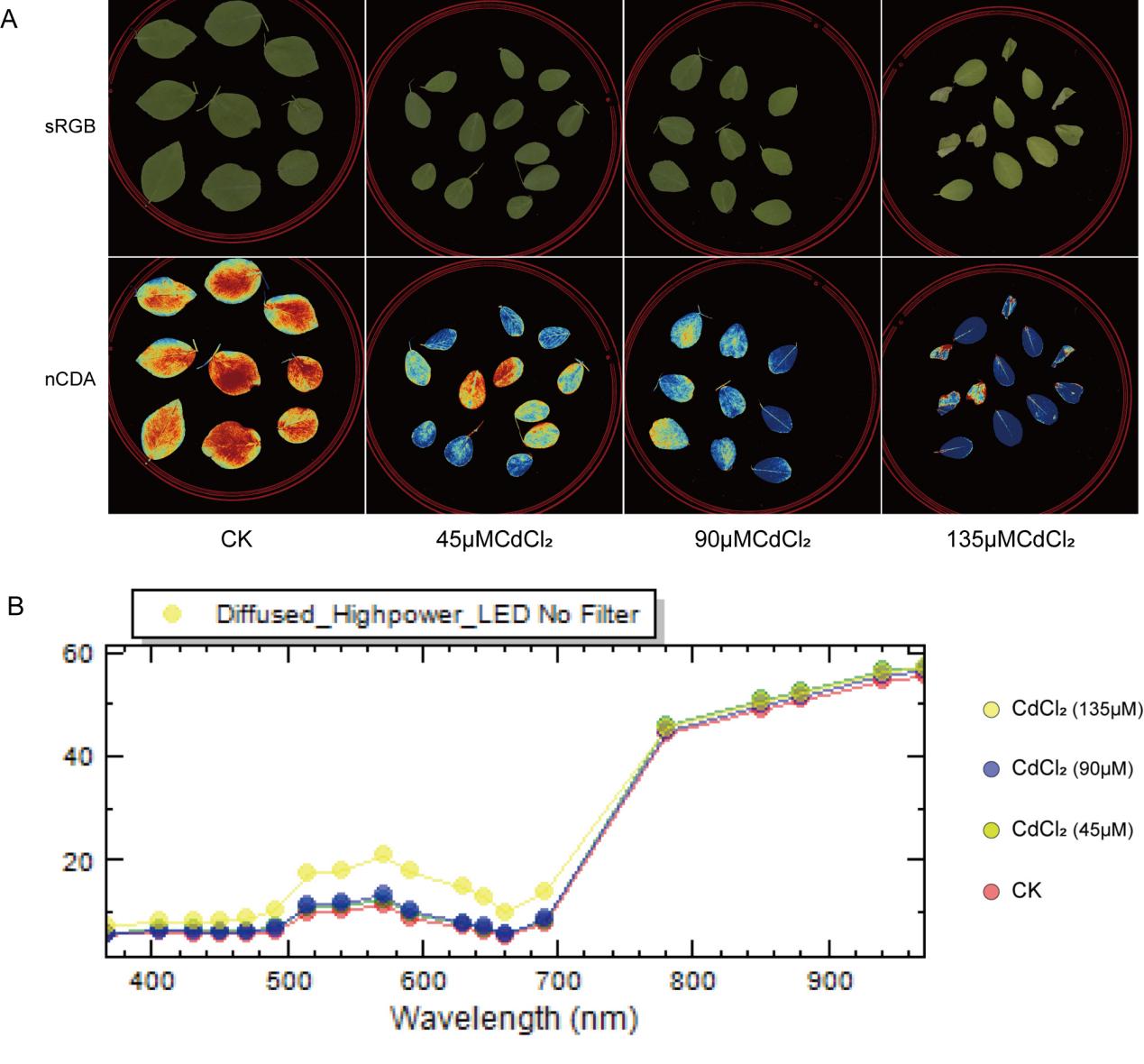
**

**Figure S1 Hyperspectral phenotyping of alfalfa leaves under cadmium (Cd) stress.**

(A) Representative leaf images under varying Cd concentrations. Top panel: sRGB images showing visible coloration. Bottom panel: Corresponding pseudocolor maps of the normalized chlorophyll a index (NCDA), where the color scale from red to blue indicates high to low chlorophyll a density, respectively.

(B) Leaf spectral reflectance profiles. The x- and y-axes represent wavelength (nm) and reflectance (%), respectively.


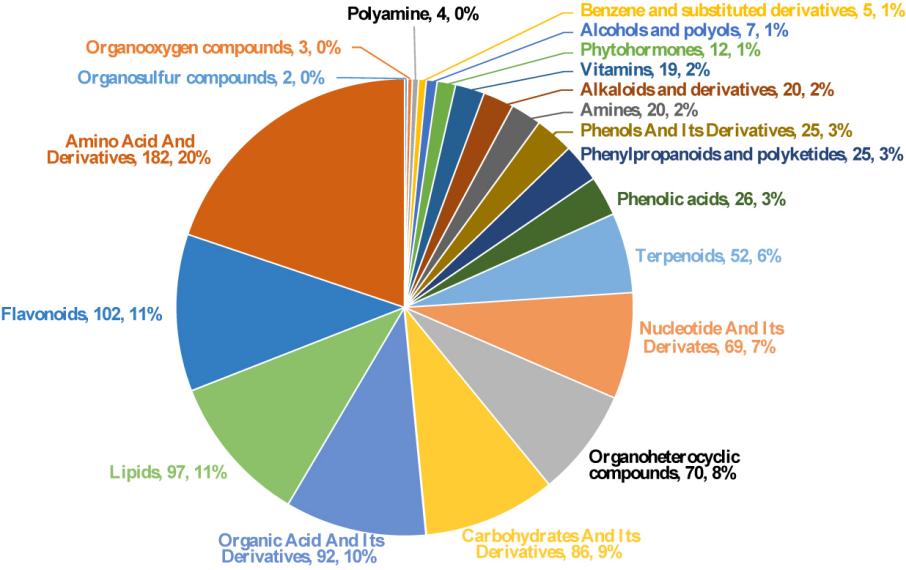


**Figure S2** Classification of identified metabolites on the basis of biosynthetic pathways. The chart displays the proportions of 918 metabolites assigned to their primary metabolic pathways.


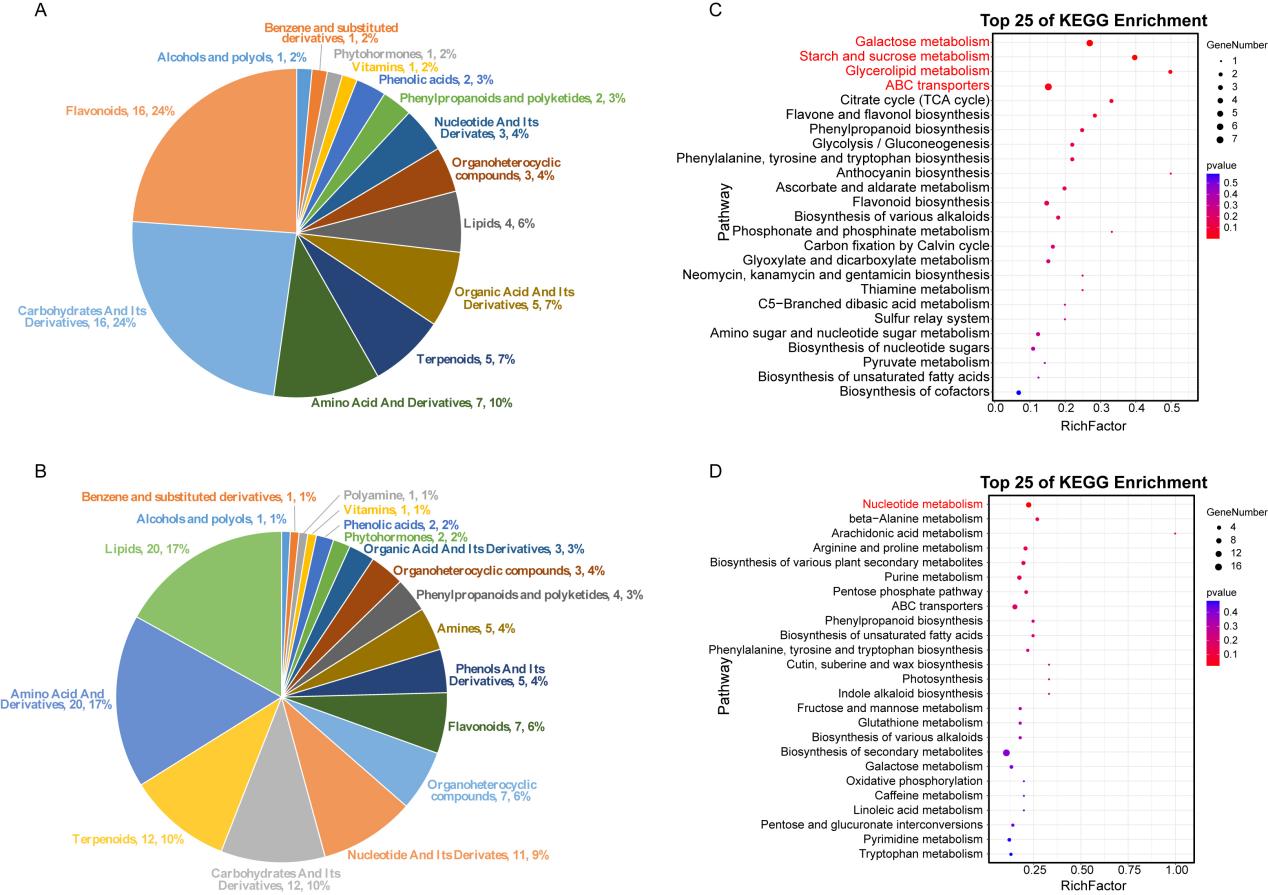


**Figure S3** Classification and KEGG pathway analysis of DAMs associated with Cd-responsive genes. (A) Proportion of metabolite classes upregulated. (B) Proportion of metabolite classes downregulated. (C) KEGG enrichment of upregulated metabolites. (D) KEGG enrichment of downregulated metabolites.


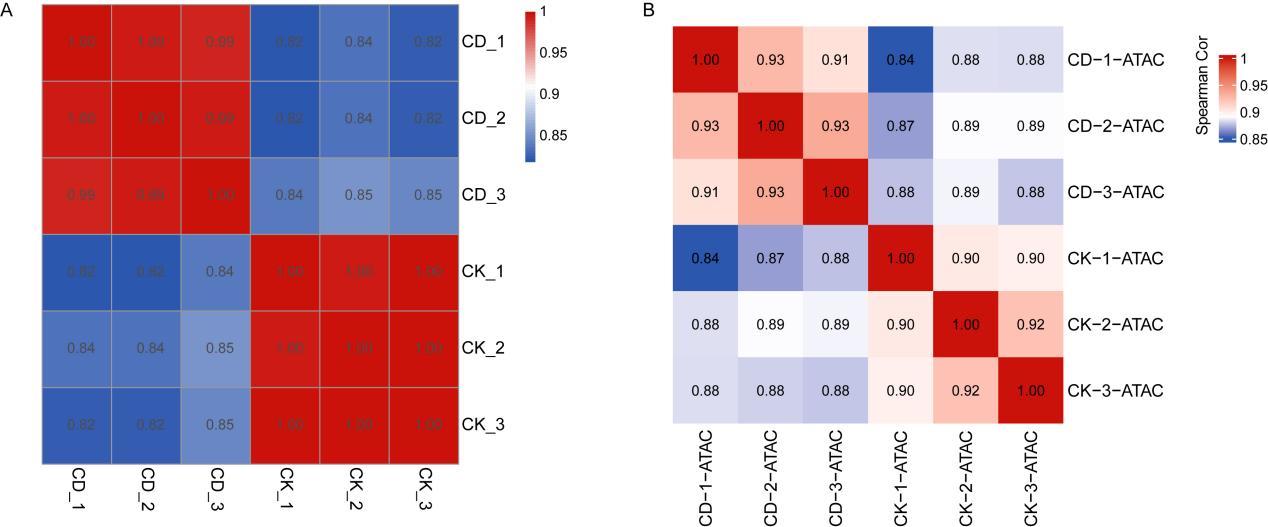


**Figure S4** Intersample correlation between control and treatment groups. (A) RNA-seq intersample correlation. (B) ATAC-seq intersample correlation.


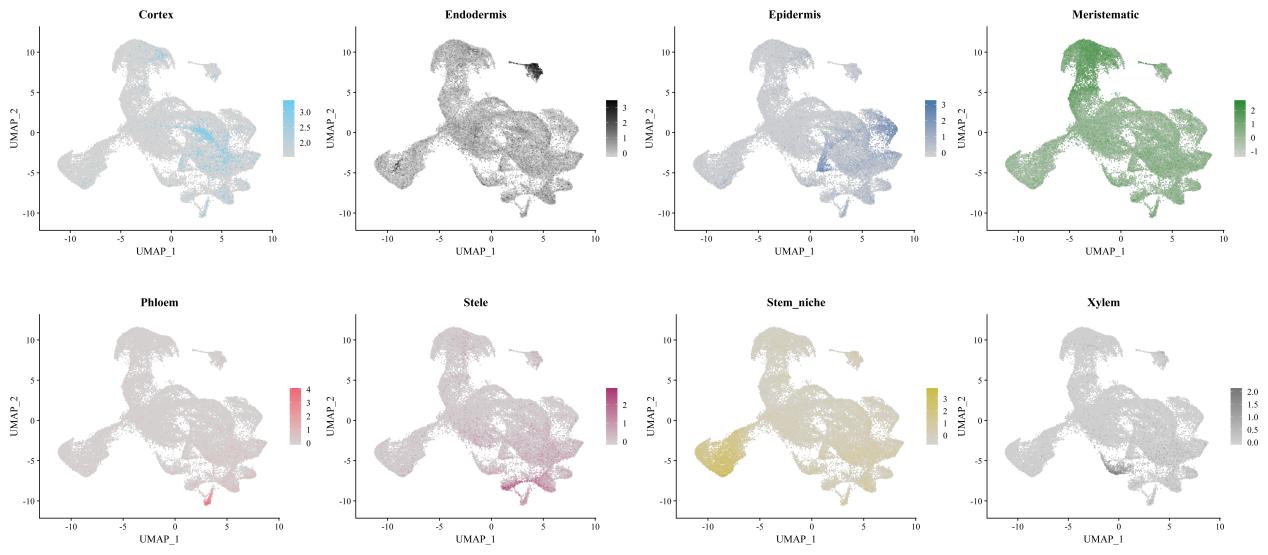


**Figure S5** UMAP visualization of eight root cell type identities. The annotated cell types include the cortex, endodermis, epidermis, meristematic, phloem, stele, stem niche, and xylem.


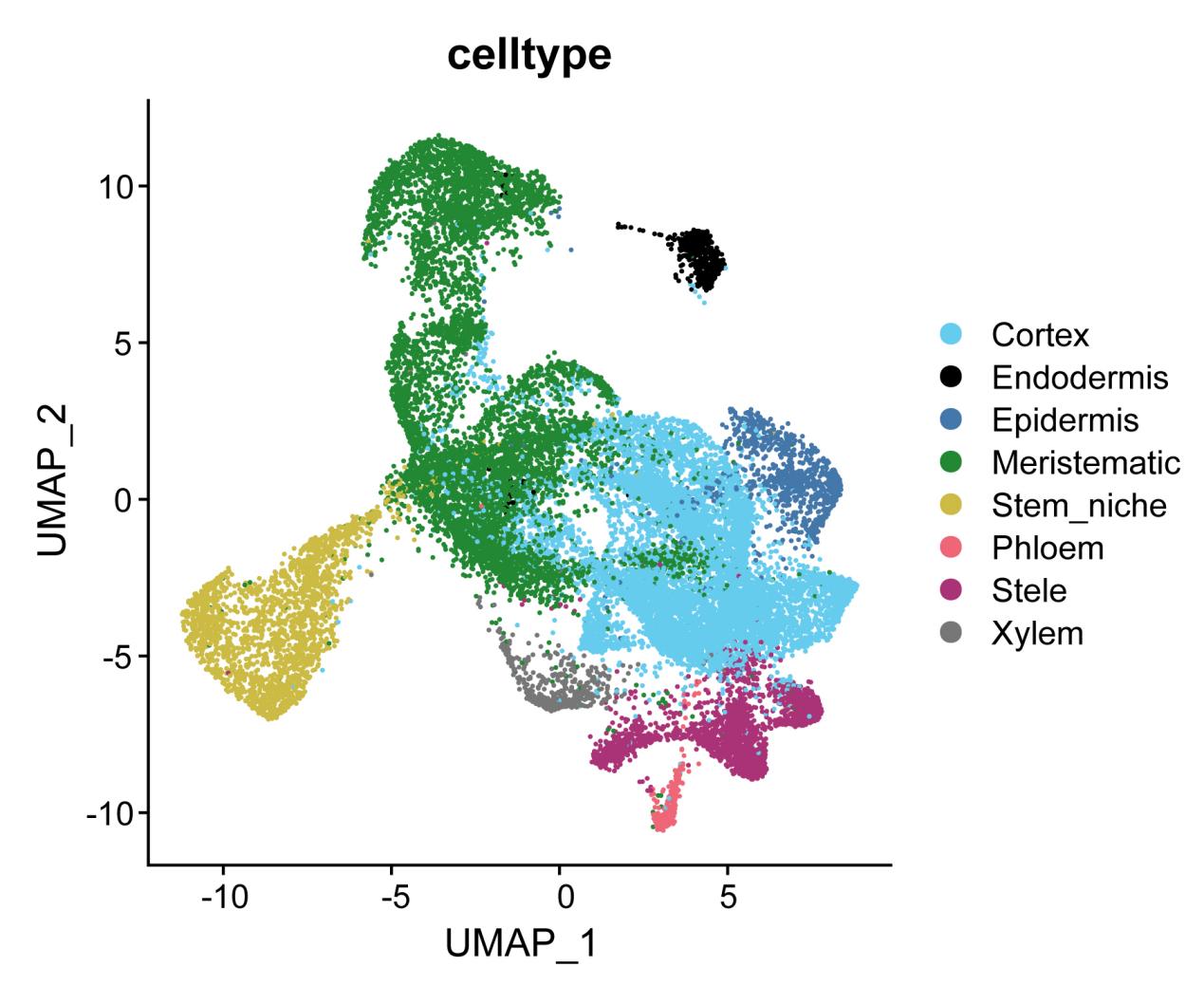


**Figure S6** UMAP visualization of cell clusters in alfalfa root tips under cadmium stress.


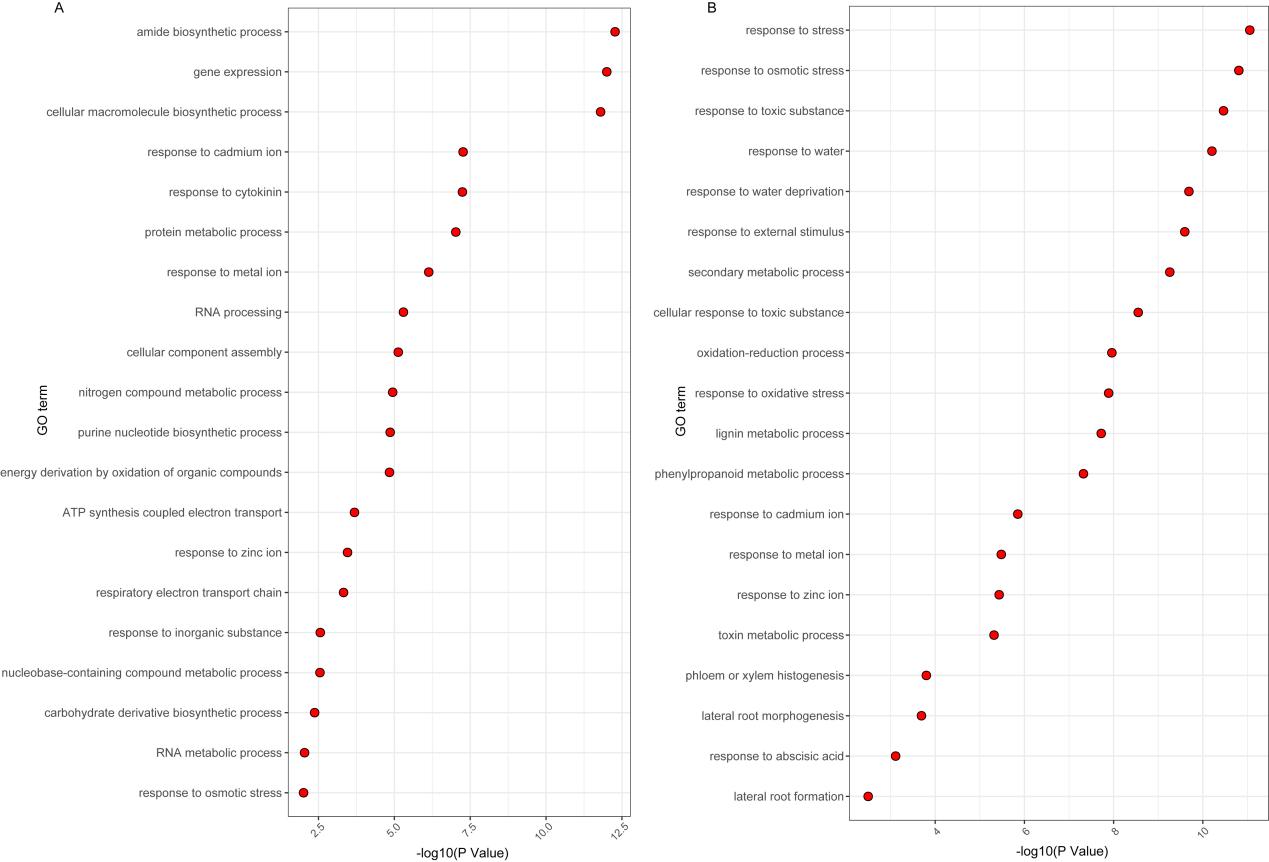


**Figure S7** Gene Ontology functional enrichment analysis of cadmium-induced differentially expressed genes. (A) Biological processes enriched for upregulated DEGs. (B) Biological processes enriched among downregulated DEGs.


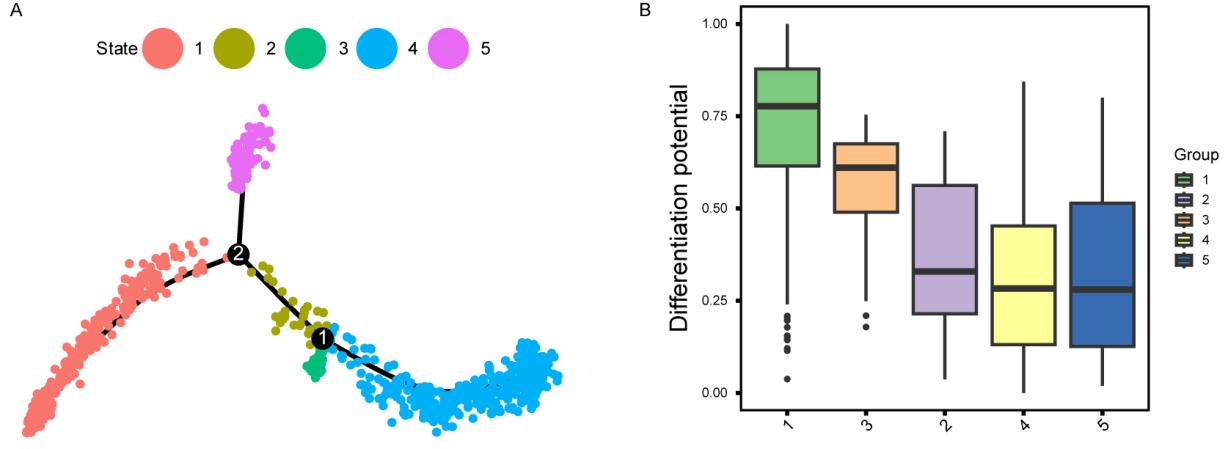


**Figure S8** Pseudotemporal ordering and differentiation potential of the endodermis.

1. Five distinct cell states identified along the pseudotime trajectory.

(B) Predicted differentiation potential for each state using CytoTRACE


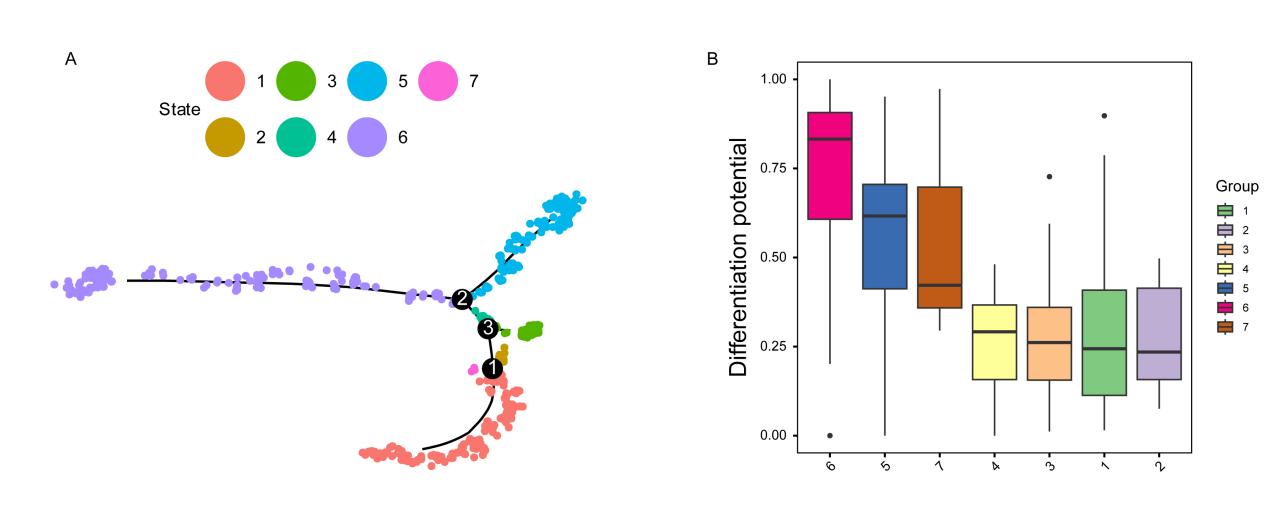


**Figure S9** Pseudotemporal ordering and differentiation potential of phloem cells.

1. Seven distinct cell states were identified along the pseudotime trajectory.

(B) Differentiation potential of each state predicted by CytoTRACE.


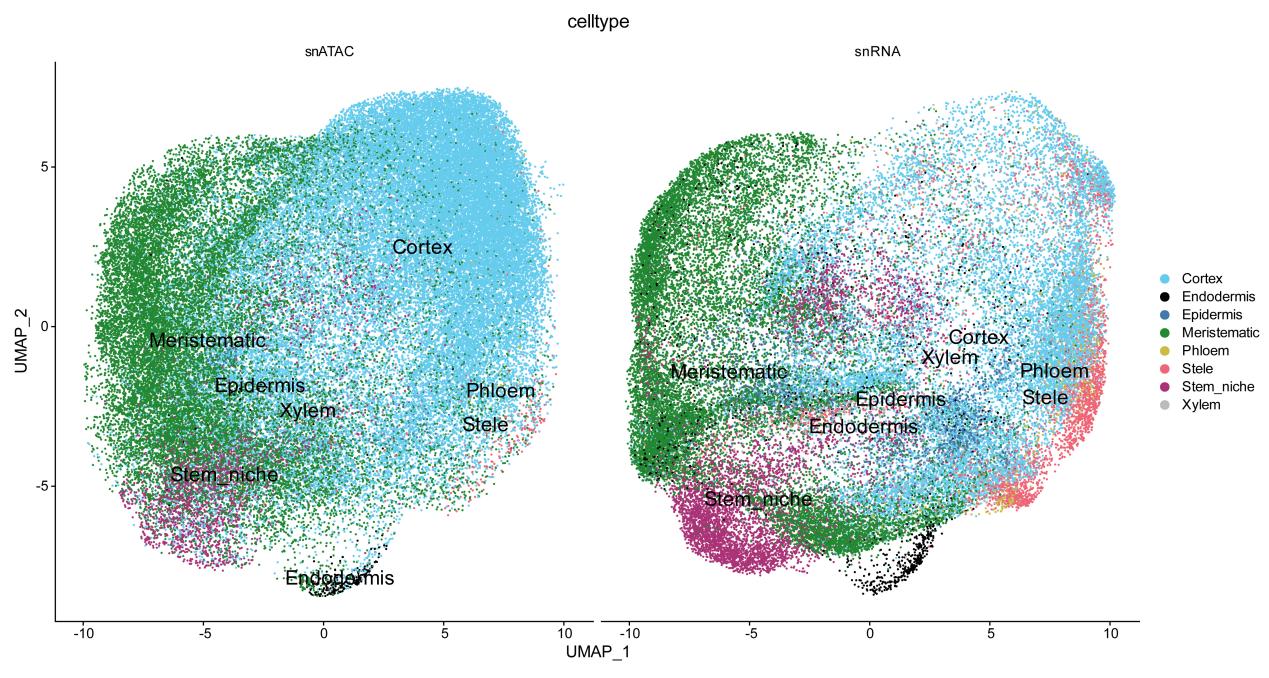


**Figure S10** A single-cell atlas of the alfalfa root defined by multimodal snRNA-seq and snATAC-seq analysis. UMAP visualization shows distinct cell type identities.


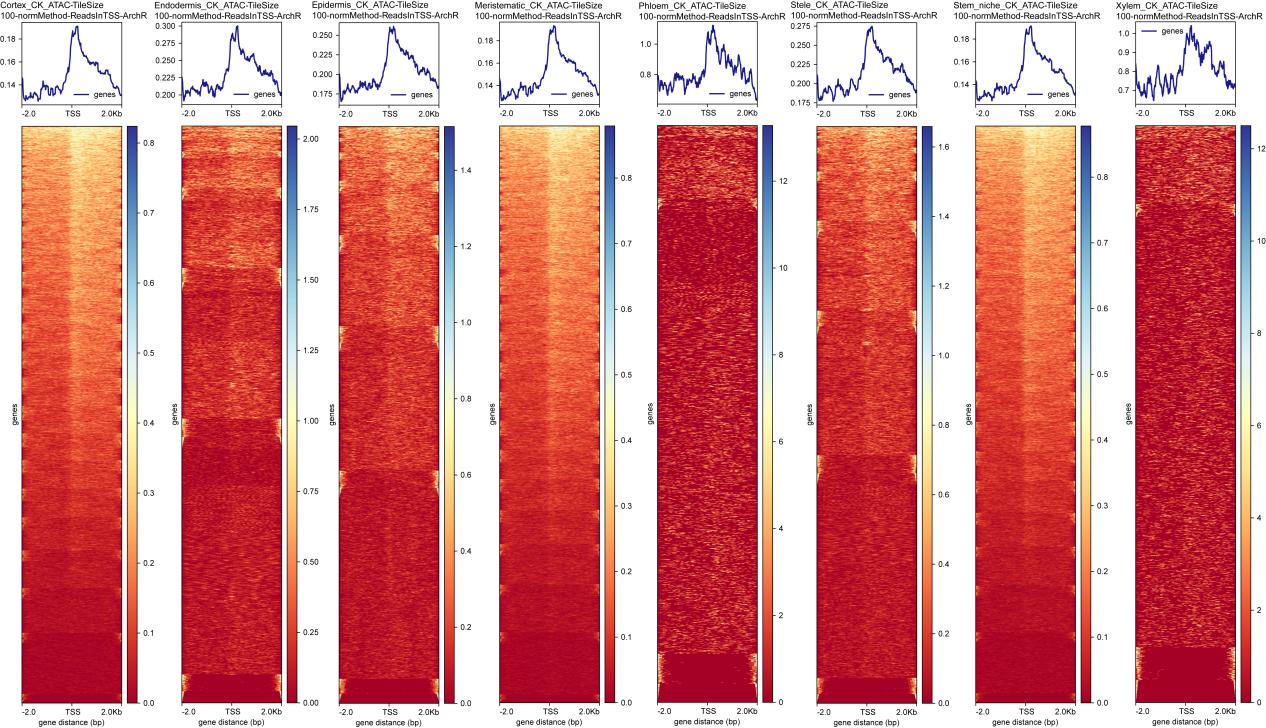


**Figure S11** Variation in ATAC-seq signals in eight identified cell types.


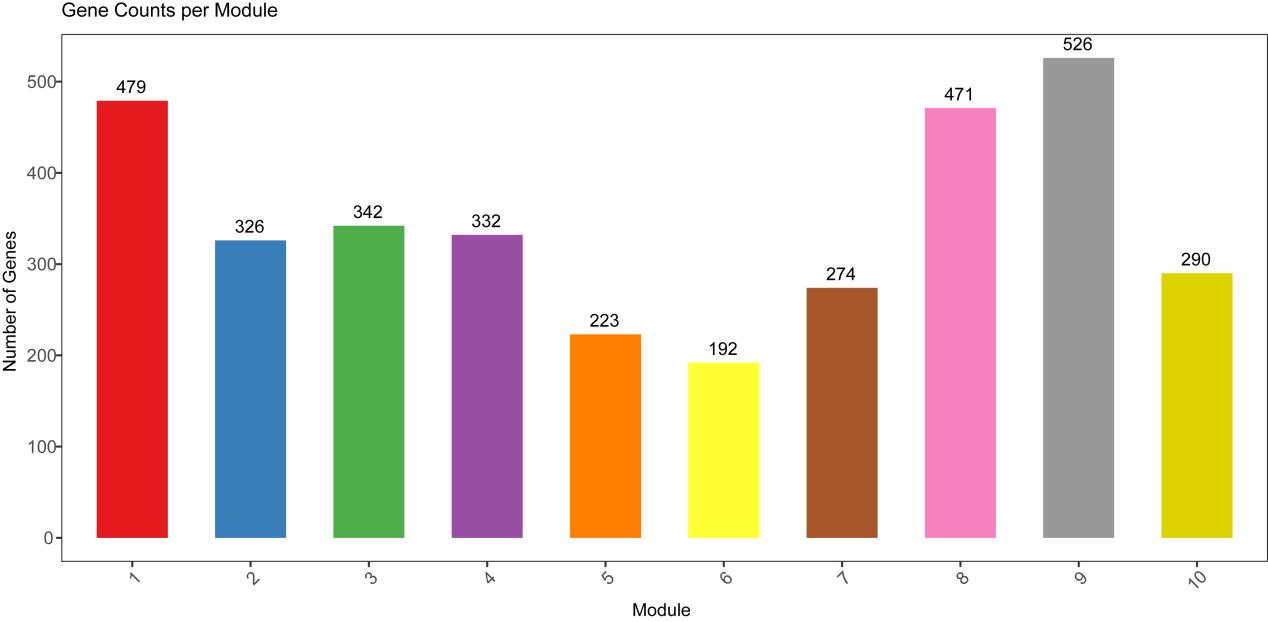


**Figure S12** Number of genes in different coexpression modules.


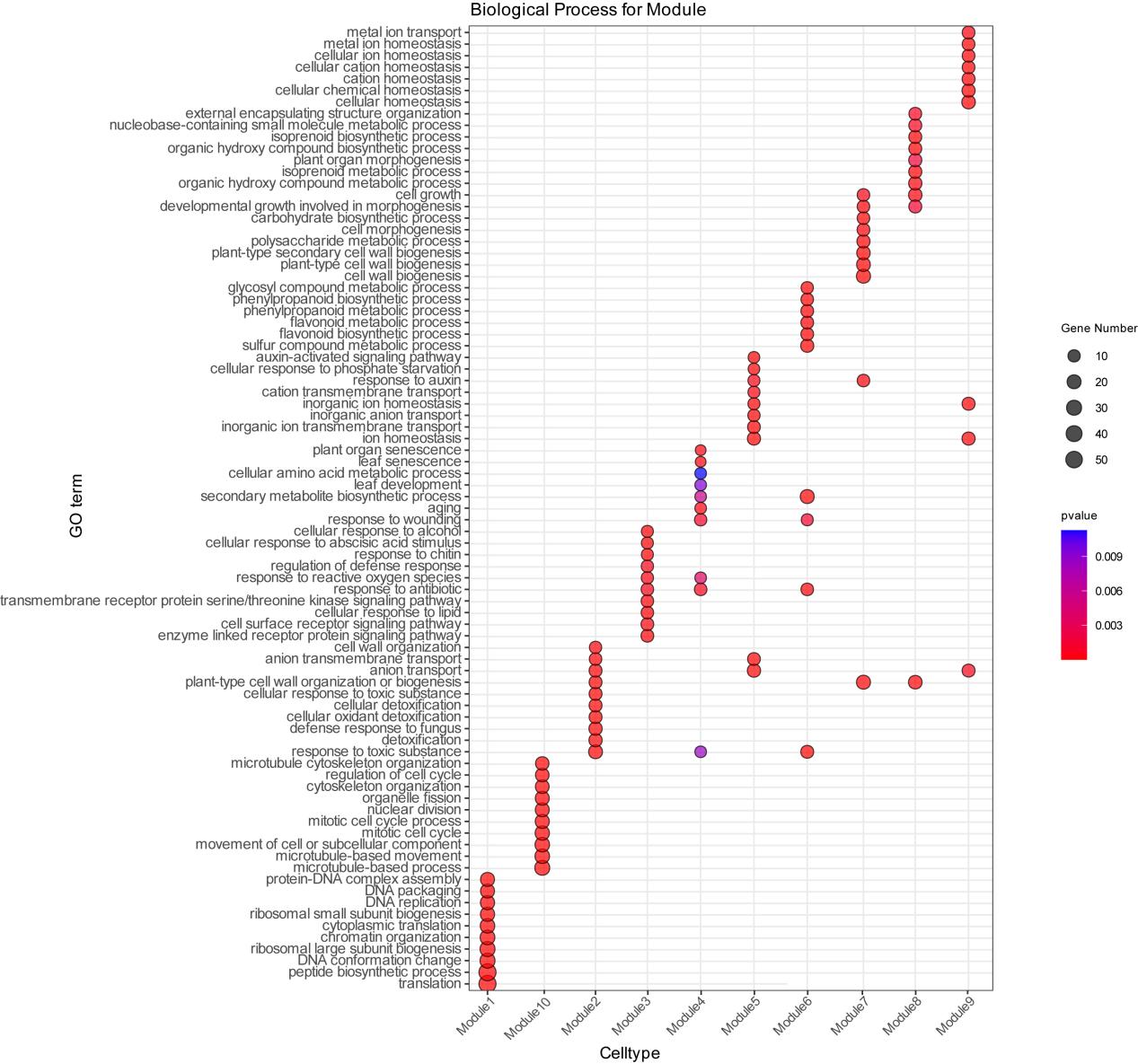


**Figure S13** Functional enrichment analysis of different coexpression modules.


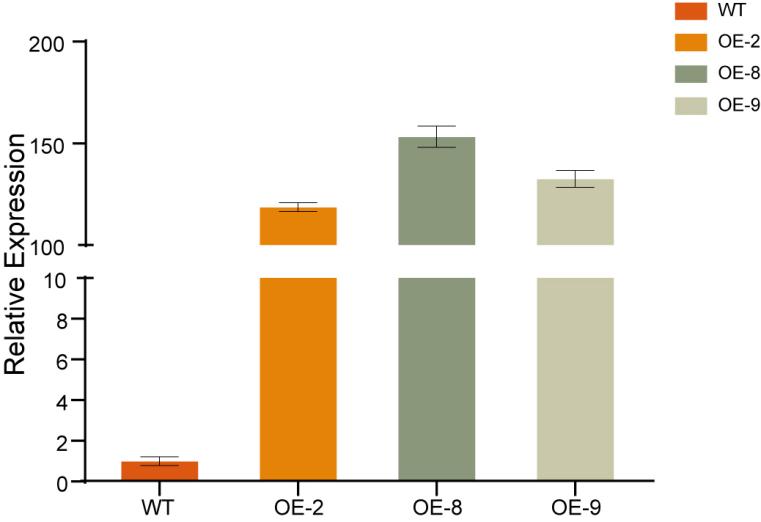


**Figure S14** Relative expression levels of *MsCML*genes in overexpression lines.


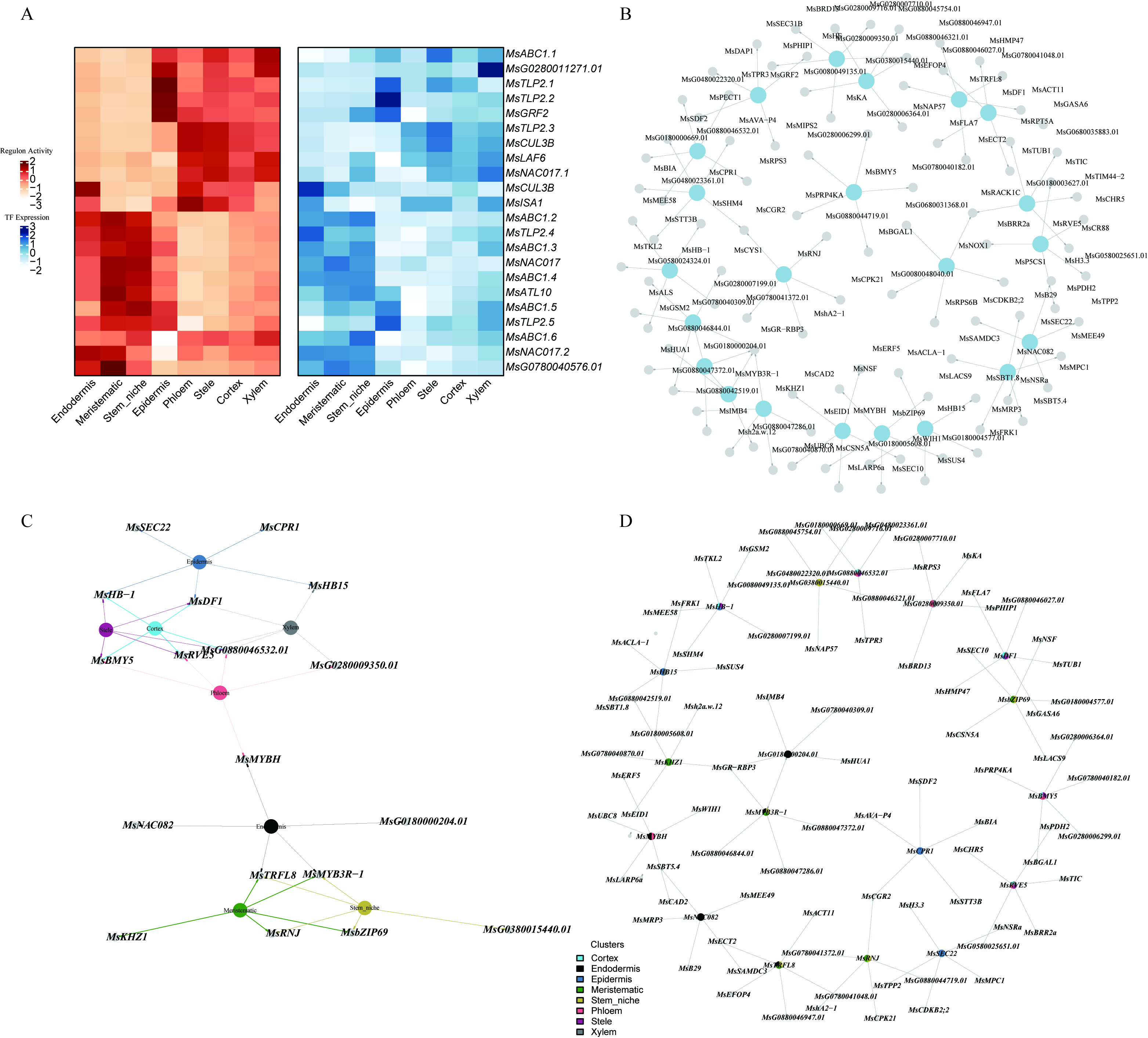


**Figure S15** Identification of cell type-specific transcription factor-target regulatory modules.

1. Correlation between transcription factor expression across cell types (snRNA-seq) and regulatory activity (motif enrichment based on snATAC-seq). Rows correspond to selected transcription factors; columns correspond to cell clusters.

(B) Gene regulatory networks illustrating each TF's regulatory network. Core nodes (central nodes): represent the most representative transcription factor in each cell cluster. Peripheral nodes: represent direct target genes regulated by these TFs.

(C) Top 5 most representative transcription factor (TF) regulons identified by SCENIC in each cell type.

(D) Interaction relationships between transcription factors (TFs) and their downstream target genes across different cell types. Core nodes (central nodes): represent the most representative transcription factor in each cell cluster. Peripheral nodes: Directly regulated target genes controlled by these TFs. Pie chart: If a transcription factor is identified across multiple cell clusters, its node is visualized as a pie chart showing its proportion across different cell types.


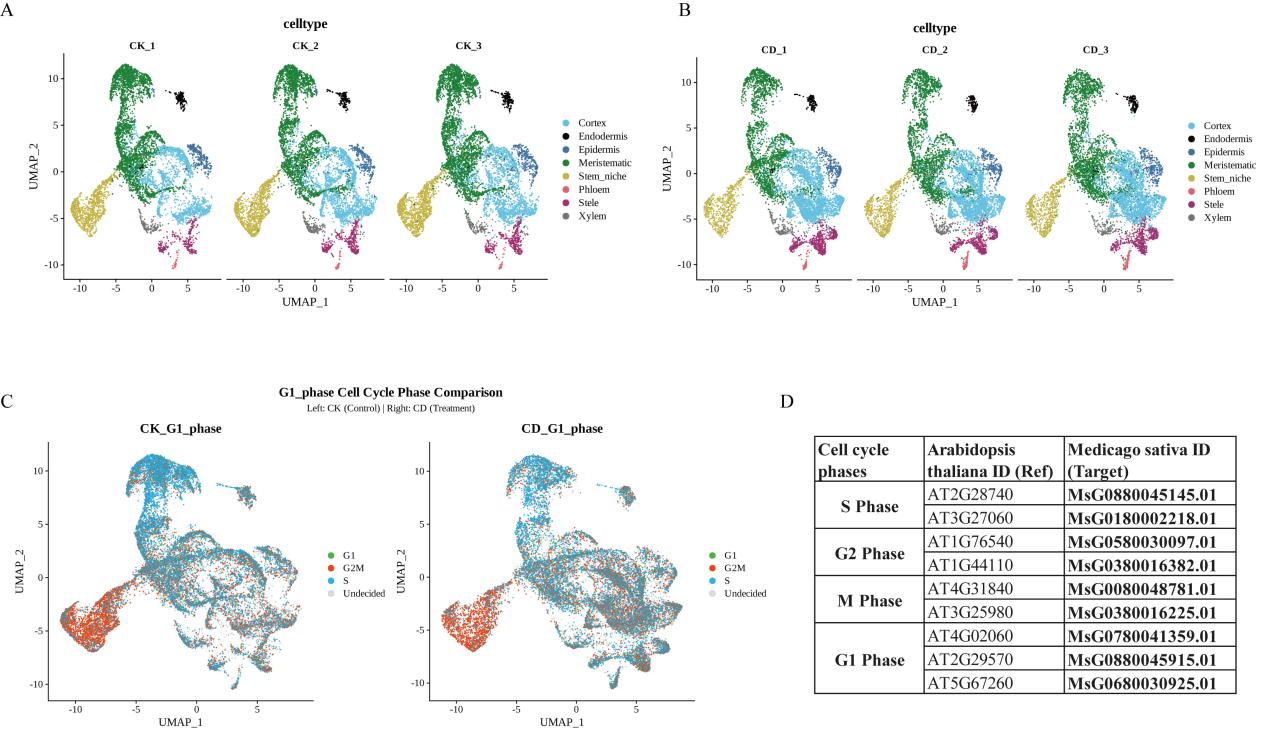


**Figure S16** Quality control and assessment of technical artifacts in single-cell RNA sequencing data under cadmium stress.

(A, B) UMAP visualization of all cells from control (A) and cadmium-treated (B) samples. Each condition includes three biological replicates, and the consistent clustering patterns across replicates confirm that the observed changes in cell type proportions reflect reproducible biological responses rather than technical noise.

(C) UMAP projection of cells colored by cell cycle phase (G1, S, G2/M). The similar distribution of cell cycle phases between the control and cadmium-treated groups indicates that cell cycle variation did not significantly contribute to the differences in cell type composition.

(D) List of gene IDs for canonical cell cycle markers used for cell cycle scoring

**Table S1** Marker gene information for various cell types within the root system of alfalfa.

**Table S2** Detailed information on the differentially abundant metabolites (DAMs) under Cd stress.

**Table S3** Proportion of cell types in alfalfa roots under normal and cadmium stress conditions.

**Table S4** Cadmium exposure-induced upregulated genes in alfalfa at single-cell resolution.

**Table S5** Cadmium exposure-induced downregulated genes in alfalfa at single-cell resolution.

**Table S6** Clustering of gene expression patterns in the endodermis based on pseudotime analysis.

**Table S7** Clustering of gene expression patterns in phloem based on pseudotime analysis.

**Table S8** Differential peaks of chromatin accessibility in alfalfa under cadmium stress.

**Table S9** Ten distinct coexpression modules related to the Cd response in alfalfa root cell types.

**Table S10** Differentially expressed genes associated with the MsMT2A coexpression network.

**Table S11** Differentially expressed genes associated with the MsHMP47 coexpression network.

**Table S12** Differentially expressed genes associated with the MsCML coexpression network.

**Table S13** Spectral reflectance of alfalfa (Medicago sativa) leaves under different cadmium (Cd) concentrations (%).
