# Supplementary material for: Single-nucleus RNA-seq and ATAC-seq analyses provide molecular insights into cadmium-stress response in alfalfa roots
Source: Hortic Res. 2026 Apr 6;13(8):uhag117. doi: 10.1093/hr/uhag117 (PMC13392044; doi:10.1093/hr/uhag117)
Supplement: Web_Material_uhag117 [file web_material_uhag117.zip › Supplementary Method S1.docx]

**Supplementary Method S1**

**2.13 Yeast transformation**

**2.13.1 Transformation procedure**

The recombinant plasmids pYES2-NTB-MsGSH1, pYES2-NTB-MsABCC2, pYES2-NTB-MsMT2A, and pYES2-NTB-MsHMP47, along with the empty vector controls pyes2-NTB (ycf1) and pyes2-NTB (BY4741), were individually introduced into the yeast recipient strain ycf1. The detailed transformation procedure was as follows: A single colony of ycf1 from a YPDA plate was inoculated into 4 mL of YPDA liquid medium and cultured overnight at 30°C with shaking at 225 rpm until the OD600 exceeded 1.5. An aliquot of the overnight culture was transferred to 50 mL of fresh YPDA medium to an initial OD600 of 0.2, followed by incubation under the same conditions until the OD600 reached approximately 0.6. The cells were harvested by centrifugation at 4000 rpm for 5 minutes. The pellet was sequentially resuspended and washed with 20 mL of sterile water and 5 mL of 0.1 M LiAc. Finally, the cell pellet was resuspended in 500 μL of 0.1 M LiAc and aliquoted into 1.5 mL microcentrifuge tubes (50 μL per transformation).

For each transformation reaction, the following components were added to a 50 μL aliquot of competent cells: 240 μL of 50% PEG3350, 36 μL of 1 M LiAc, 5 μL of ssDNA (20 mg/mL), and 5 μL of plasmid DNA. The mixture was thoroughly combined by pipetting or vigorous vortexing. It was then incubated in a water bath at 30°C for 30 minutes, subjected to heat shock at 42°C for 25 minutes, and allowed to recover at 30°C for 30 minutes. After recovery, the cells were collected by centrifugation, the supernatant was discarded, and the pellet was resuspended in 200 μL of sterile water. The entire suspension was spread onto SD-Ura dropout selection plates and incubated at 30°C for 3 to 5 days.

**2.13.2 Phenotypic verification**

To assess the cadmium tolerance of the transformants, SG-Ura solid media (with galactose replacing glucose) containing varying concentrations of CdCl₂ (0, 50, 80, 120, 150, and 200 μM) were prepared. Single colonies from each experimental and control group that were previously verified by PCR were picked and suspended in medium to an OD600 of 0.05. These suspensions were then serially diluted (10⁰, 10⁻¹, 10⁻²), and spot assays were performed by spotting each dilution onto cadmium-containing plates. The plates were incubated at 30°C, and colony growth was documented after 3 to 7 days of cultivation.

**Supplementary Method S2**

**2.15 qRT–PCR analysis of transgenic plants**

T3 seeds of the transgenic Arabidopsis lines were stored at the laboratory of the Institute of Animal Science of the Chinese Academy of Agricultural Sciences. For plant growth, surface-sterilized seeds were stratified at 4°C for 2 days to synchronize their germination. The seeds were subsequently cultivated on 1/2 MS medium supplemented with **60 mg/L kanamycin (for selection of positive transgenic plants)** under a 16/8 h light/dark photoperiod at 24/20°C (day/night) in a growth chamber. After 10–14 days, healthy seedlings were transplanted into soil and grown under the same conditions for an additional 3–4 weeks. Total RNA was extracted from **Arabidopsis leaf tissues** using TRIzol reagent according to the manufacturer's instructions. Gene-specific primers for MsCML70 were designed using Primer 5.0 software: the forward primer was MsCML70F (5'-CTATTGAAGCAGTGGATT-3'), and the reverse primer was MsCML70R (5'-TAGACTTAGACTCACCCA-3'). **The Arabidopsis Actin (AtActin) gene was used as an internal control with a forward primer (5'-GGTAACATTGTGCTCAGTGGTGG-3') and a reverse primer (5'-AACGACCTTAATCTTCATGCTGC-3').** qRT–PCR was performed using SYBR Premix Ex Taq (TaKaRa, Japan) on a 7500 Real-Time PCR System (Applied Biosystems, USA). Each reaction was performed with three technical replicates, and the expression level was normalized to that of the **AtActin** gene [70]. The relative gene expression levels were calculated using the comparative 2–ΔΔCT method [71]. qRT–PCR analysis confirmed the overexpression of MsCML70 in the transgenic lines (Fig. S14). On the basis of the results, three T3-homozygous lines exhibiting high expression levels were selected for subsequent phenotypic analyses.
